# *In vitro* antiproliferative, anti-inflammatory effects and molecular docking studies of natural compounds isolated from *Sarcocephalus pobeguinii* (Hua ex Pobég)

**DOI:** 10.3389/fphar.2023.1205414

**Published:** 2023-06-21

**Authors:** Emmanuel Mfotie Njoya, Brigitte Ndemangou, Jude Akinyelu, Aristide M. Munvera, Chika. I. Chukwuma, Pierre Mkounga, Samson S. Mashele, Tshepiso J. Makhafola, Lyndy J. McGaw

**Affiliations:** ^1^ Centre for Quality of Health and Living, Faculty of Health and Environmental Sciences, Central University of Technology, Bloemfontein, South Africa; ^2^ Department of Paraclinical Sciences, Faculty of Veterinary Science, University of Pretoria, Pretoria, South Africa; ^3^ Department of Biochemistry, Faculty of Science, University of Yaoundé I, Yaoundé, Cameroon; ^4^ University Institute of Technology of Wood Technology, Mbalmayo, Cameroon; ^5^ Department of Biochemistry, Federal University Oye-Ekiti, Oye, Nigeria; ^6^ Department of Organic Chemistry, Faculty of Science, University of Yaoundé I, Yaound, Cameroon

**Keywords:** Sarcocephalus pobeguinii, hederagenin, inflammation, cancer, cytotoxicity, selective index, apoptosis, docking score

## Abstract

**Background:** *Sarcocephalus pobeguinii* (Hua ex Pobég) is used in folk medicine to treat oxidative-stress related diseases, thereby warranting the investigation of its anticancer and anti-inflammatory properties. In our previous study, the leaf extract of *S. pobeguinii* induced significant cytotoxic effect against several cancerous cells with high selectivity indexes towards non-cancerous cells.

**Aim:** The current study aims to isolate natural compounds from *S. pobeguinii*, and to evaluate their cytotoxicity, selectivity and anti-inflammatory effects as well as searching for potential target proteins of bioactive compounds.

**Methods:** Natural compounds were isolated from leaf, fruit and bark extracts of *S. pobeguinii* and their chemical structures were elucidated using appropriate spectroscopic methods. The antiproliferative effect of isolated compounds was determined on four human cancerous cells (MCF-7, HepG2, Caco-2 and A549 cells) and non-cancerous Vero cells. Additionally, the anti-inflammatory activity of these compounds was determined by evaluating the nitric oxide (NO) production inhibitory potential and the 15-lipoxygenase (15-LOX) inhibitory activity. Furthermore, molecular docking studies were carried out on six putative target proteins found in common signaling pathways of inflammation and cancer.

**Results:** Hederagenin (**2**), quinovic acid 3-O-[α-D-quinovopyranoside] (**6**) and quinovic acid 3-O-[β-D-quinovopyranoside] (**9**) exhibited significant cytotoxic effect against all cancerous cells, and they induced apoptosis in MCF-7 cells by increasing caspase-3/-7 activity. (**6**) showed the highest efficacy against all cancerous cells with poor selectivity (except for A549 cells) towards non-cancerous Vero cells; while (**2**) showed the highest selectivity warranting its potential safety as a chemotherapeutic agent. Moreover, (**6**) and (**9**) significantly inhibited NO production in LPS-stimulated RAW 264.7 cells which could mainly be attributed to their high cytotoxic effect. Besides, the mixture nauclealatifoline G and naucleofficine D (**1**), hederagenin (**2**) and chletric acid (**3**) were active against 15-LOX as compared to quercetin. Docking results showed that JAK2 and COX-2, with the highest binding scores, are the potential molecular targets involved in the antiproliferative and anti-inflammatory effects of bioactive compounds.

**Conclusion:** Overall, hederagenin (**2**), which selectively killed cancer cells with additional anti-inflammatory effect, is the most prominent lead compound which may be further investigated as a drug candidate to tackle cancer progression.

## 1 Introduction

Cancer is one of the leading causes of death worldwide, accounting 19.3 million new cancer cases and about 10 million deaths in 2020 (Sung et al., 2021). This chronic disease is characterized by abnormal and uncontrolled proliferation of cancer cells which later invades normal tissues and organs, and eventually spreading throughout the body. Several reports have proven the cross-talk between inflammation and cancer due to the fact that common signaling pathways are modulated in both deleterious ailments (Hibino et al., 2021; Zhao et al., 2021). Regardless to the cancer type, inflammation conditions have been considered as one of the key factors that promotes all stages of tumorigenesis. Inflammation also helps the survival of malignant cells, therefore impeding the immune surveillance or altering the efficacy of chemotherapeutic agents (Mantovani et al., 2008; Peczek et al., 2022). As one of the most important cancer treatment strategies, chemotherapy uses powerful drugs to kill fast-growing cells, but it induces substantial side effects due to the fact that normal cells are also affected (Amjad et al., 2022). To date, great progress has been made for the discovery and development of effective and safe chemotherapeutic agents, however the number of cancer-related deaths increases every day. This situation urgently requires the search of alternative treatments to improve the quality of life of patients. Natural therapies including medicinal plants constitute a reservoir for the discovery of new anticancer lead compounds. Additionally, as above-mentioned, chronic inflammation accelerates cancer progression, and it has been proven that some anti-inflammatory drugs such as aspirin, celecoxib, diclofenac, etc. are being used against cancer (Rayburn et al., 2009; Zappavigna et al., 2020; Lai et al., 2022). As such, controlling inflammation may represent a valid strategy for cancer prevention and therapy. Thus, developing anticancer agents with additional anti-inflammatory effect is the best approach to tackle cancer progression.

Cameroon has a rich biodiversity, with about 8,620 plants species which are used by local population for the treatment of several ailments (Kuete and Efferth, 2010; Ntie-Kang et al., 2013). Among these plants, *Sarcocephalus pobeguinii* (Hua ex Pobég) (synonym of *Nauclea pobeguinii* (Hua ex Pobég) Merr.) is used as infusion or decoction in the treatment of fever, malaria, stomach-ache, sexual and reproductive dysfunctions, epilepsy, diabetes mellitus, hypertension, infectious diseases and jaundice (Mesia et al., 2005; Jiofack et al., 2009; Karou et al., 2011; Kuete et al., 2015; Haudecoeur et al., 2018). In a study investigating the cytotoxicity of selected Cameroonian medicinal plants, the bark extract of *S. pobeguinii* and its isolated compounds (resveratrol and a glucoside derivative) exhibited antiproliferative potential against multi-factorial drug-resistant cancer cell lines (Kuete et al., 2015). In our previous study, the antiproliferative effect of different plant parts (leaf, fruit, bark and root) extracts of *S. pobeguinii* were evaluated on several human cancer cell lines, and leaf extract induced significant cytotoxic effect with high selectivity indexes (Mfotie Njoya et al., 2017). So far, phytochemical investigations carried out on bark and root extracts of *S. pobeguinii* have resulted in the isolation and characterization of compounds such as strictosamide, 5-carboxystrictosidine, methylangustoline, 3-O-β-D-fucosyl-quinovic-acid, 3-keto-quinovic-acid; 19-O-methylangustoline, 3-acetoxy-11-oxo-urs-12-ene, p-coumaric acid, citric acid trimethyl ester, resveratrol, resveratrol β-D-glucopyranoside (Karou et al., 2011; Kuete et al., 2015; Yüce et al., 2019). Despite the use of leaves and fruits of *S. pobeguinii* in traditional medicine, the isolation of compounds from these plant parts as well as their biological effects have not yet been investigated to the best of our knowledge. Moreover, the use of leaves and fruits for medicinal purposes instead of roots and barks is advantageous for the conservation and sustainable maintenance of medicinal plants. Therefore, the current work aims to find bioactive compounds from leaf and fruit extracts of *S. pobeguinii* and to evaluate their efficacy, selectivity and anti-inflammatory effects. In addition, this study has been also extended to the isolation of active constituents from roots of *S. pobeguinii* which might also yield to the discovery of new anticancer lead compounds.

## 2 Materials and methods

### 2.1 Plant material

Different parts (leaves, fruits, and barks) of *S. pobeguinii* were harvested in Ezezan (Nyom II), a neighbouring locality situated at 40 km from Yaoundé (Cameroon). A voucher specimen was prepared and the authentication was done by Mr. Ngansop Eric, a plant taxonomist, after comparison with the specimen number N°32,567 BRF/CAM already available in the library of the National Herbarium of Cameroon. Our plant material was then registered under the number Letouzey R.12493 (YA).

### 2.2 Preparation of extracts and isolation of compounds

The powder (907 g) obtained from the air dried and grounded fruits of *S. pobeguinii* was macerated in the mixture of methanol and methylene chloride (1:1) at room temperature for 48 h with occasional stirring, and the crude extract was obtained after filtration with Whatman N⁰1, followed by evaporation of the solvent to dryness *in vacuo*. The dried extract (76.5 g) was re-dissolved in MeOH and partitioned with ethyl acetate (EtOAc) to yield the EtOAc fraction (10.2 g) and the MeOH residue (54.7 g). The MeOH soluble fraction was then subjected to silica gel column chromatography (CC) eluted with a gradient mixture of *n-*hexane-EtOAc (9:1; 7:3; 6:4; 0:1). The collected sub-fractions were pooled based on their thin layer chromatography (TLC) profiles and yielded six sub-fractions (F1-F6) after evaporation. Sub-fraction F_3_ (8 g) was submitted to silica gel CC, and eluted with the mixture of *n*-hexane-EtOAc (2:1) to yield the following compounds: (**1**) (6 mg) identified as a mixture of nauclealatifoline G and naucleofficine D (Agomuoh et al., 2013); (**2**) (7 mg) known as hederagenin (Joshi et al., 1999); and (**3**) (6 mg) characterized as chletric acid (Takahashi and Takani, 1978).

The powder of air-dried leaves of *S. pobeguinii* (1 kg) was extracted in MeOH and heated at 40°C–50°C for 2 h. The methanolic extract was concentrated *in vacuo* to yield a dark greenish mass (66 g) which was partitioned with *n*-hexane. The n-hexane soluble part and the methanolic residue were evaporated under pressure to obtain respectively a black residue (26.5 g) and a greenish brown extract (37.3 g). The latter was subjected to silica gel flash chromatography, and eluted respectively with CH_2_Cl_2_; CH_2_Cl_2_/MeOH (1:1) and MeOH. The *n*-hexane soluble part and the CH_2_Cl_2_ fraction were mixed due to their similar TLC profiles, and this mixed fraction was submitted to silica gel CC eluted with the gradient mixture of *n*-hexane and EtOAc (9:1; 3:1; 3:2; 1:1; 1:3) to afford compound (**4**) (5 mg) identified as taraxerol (Yen et al., 2013) collected in *n*-hexane/EtOAc (3:1); compound (**5**) (4 mg) known as *α*-amyrin (3β-hydroxy-urs-12-en-3-ol) (Viet et al., 2021) collected in *n*-hexane/EtOAc (1:1); and (**6**) (6 mg) known as quinovic acid 3-O-[*α*-D-quinovopyranoside] (Mohamed, 1999) collected in *n*-hexane/EtOAc (1:3).

The powder of dried bark of *S. pobeguinii* (1 kg) was extracted in MeOH and heated at 40°C–50°C for 2 h. The crude extract was obtained after filtration with Whatman N⁰1, followed by evaporation under reduced pressure to obtain a yellow gummy residue (77 g). This methanolic extract was partitioned with *n*-hexane to yield the *n*-hexane fraction (15 g) and a residual MeOH fraction (54 g). The MeOH fraction was then subjected to silica gel CC, and eluted with a gradient of *n*-hexane/EtOAc yielding the following compounds: (**7**) (4 mg) identified as erythrodiol and (**8**) (8 mg) known as quinovic acid (Fatima et al., 2002; Aktar et al., 2009) both collected in the *n*-hexane/EtOAc (9:1); then compound (**9**) (8 mg) characterized as quinovic acid 3-O-[β-D-quinovopyranoside] (Yepez et al., 1991) collected in *n*-hexane/EtOAc (3:1); and finally compound (**10**) (4 mg) identified as latifoliamide C (Agomuoh et al., 2013) collected in *n*-hexane/EtOAc (1:9).

### 2.3 Characterization and structural elucidation of isolated compounds

**FIGURE 1:**
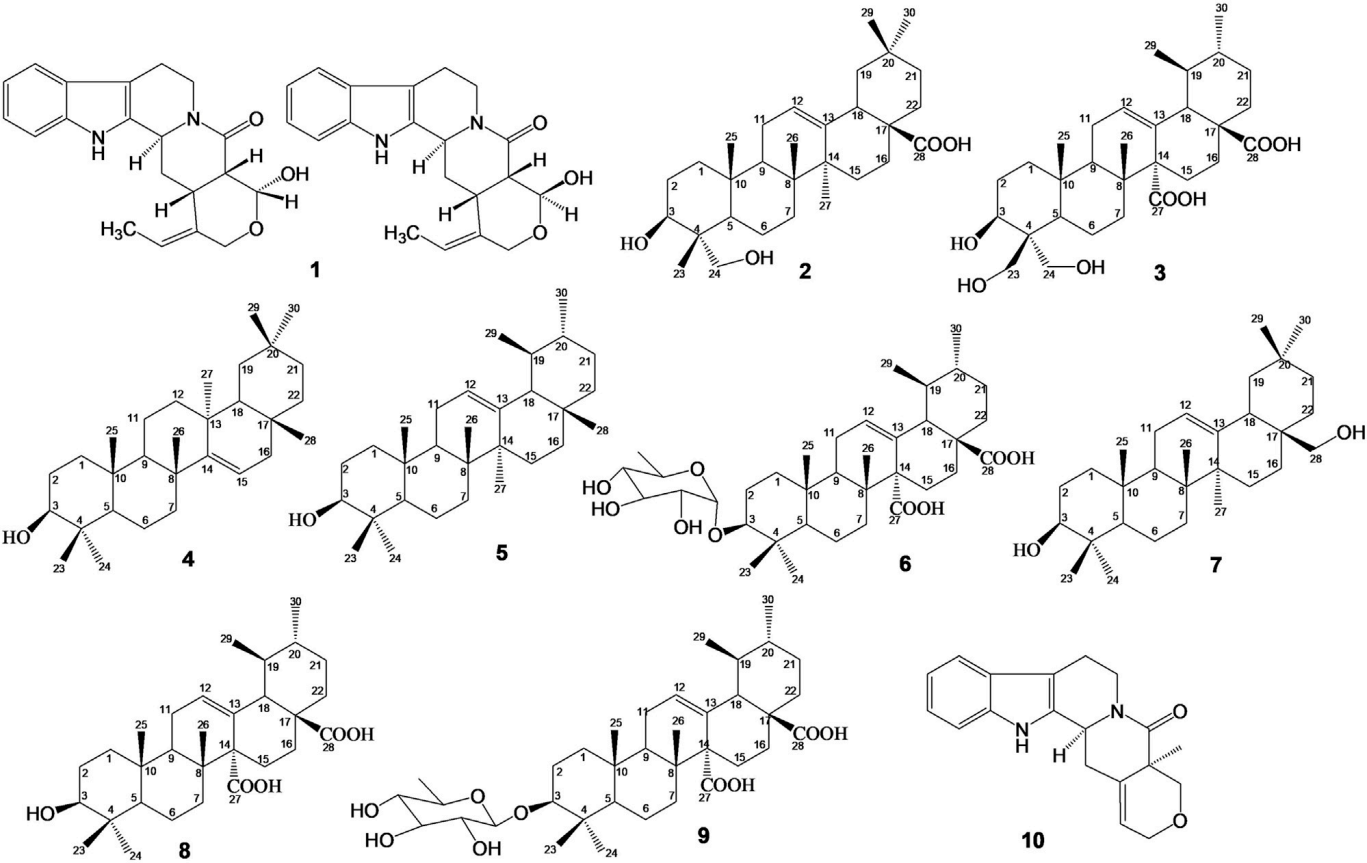
Chemical structures of isolated compounds: Mixture of Nauclealatifoline G (C20H22N2O3, 338.40 g/mol) and naucleofficine D (C20H22N2O3, 338.40 g/mol) (1), hederagenin (C30H48O4, 472.73 g/mol) (2) and chletric acid (C30H46O8, 534.69 g/mol) (3) were isolated from fruit extract of S. pobeguinii. Taraxerol (C30H50O, 426.73 g/mol) (4), α-amyrin (3β-hydroxy-urs-12-en-3-ol) (C30H50O, 426.73 g/mol) (5) and quinovic acid 3-O-[α-D-quinovopyranoside] (C36H56O9, 632.84 g/mol) (6) were isolated from leaf extract of S. pobeguinii. Erythrodiol (C30H50O2, 442.73 g/mol) (7), quinovic acid (C30H46O5, 486.69 g/mol) (8), quinovic acid 3-O-[β-D-quinovopyranoside] (C36H56O9, 632.84 g/mol) (9) and latifoliamide C (C19H20N2O2, 308.38 g/mol) (10) were isolated from bark extract of *S. pobeguinii.*

The chemical constituents of *S. pobeguinii* were purified using open silica gel column chromatography (Merck, [Darmstadt, Germany]). TLC was done on Alu g R; SIL G/UV_254_ silica gel plates (Merck, [Darmstadt, Germany]), and visualization of the spots on TLC plates was achieved either by exposure to iodine vapour, UV light or by spraying sulphuric acid and heating the plate at 75°C. Melting points were recorded on a Buchi B-54 apparatus. ^1^H and ^13^C NMR spectra as well as 2D NMR experiments (see Supplementary Material, Supplementary Figures S1–S22) were recorded in CDCl_3_, MeOH-_*d4*_, DMSO-_*d6*_ and pyridine-_*d5*_ in a JEOL ECX 500 spectrometer (Akishima, Japan) and on a Bruker ARX 400. Chemical shifts were expressed in part per million δ) relative to tetramethylsilane as internal standard. The spectroscopy data recorded were compared with data from literature, for the characterization of the chemical structures of isolated compounds which are presented in Figure 1.

### 2.4 Cell culture

Cancerous cell lines (MCF-7: human breast adenocarcinoma cells; HepG2: human hepatocellular carcinoma cells; Caco-2: human epithelial colorectal adenocarcinoma cells; A549: human epithelial lung adenocarcinoma cells), obtained from the American Type Culture Collection (ATCC) (Rockville, MD, United States of America), were cultured in Dulbecco’s Modified Eagle’s Medium (DMEM) high glucose (4.5 g/L) containing L-glutamine (4 mM) and sodium-pyruvate (Hyclone™) supplemented with 10% (v/v) fetal bovine serum (FBS) (Capricorn Scientific GmbH, South America), and incubated at 37°C with 5% CO_2_ in a humidified environment. African green monkey (Vero) kidney cells (also obtained from ATCC), a non-cancerous cell line, were grown in DMEM high glucose (4.5 g/L) containing L-glutamine (Lonza, Belgium) and supplemented with 5% FBS (Capricorn Scientific GmbH, South America) and 1% gentamicin (Virbac, RSA) in the same environment as cancer cells. The RAW 264.7 murine macrophage cells (obtained from ATCC) were cultured in DMEM high glucose (4.5 g/L) containing L-glutamine (Lonza, Belgium), supplemented with 10% FBS (Capricorn Scientific GmbH, South America) and 1% penicillin/streptomycin/fungizone (PSF) solution, and kept at 37°C in a 5% CO_2_ humidified environment.

### 2.5 Antiproliferation assay

The cancerous and non-cancerous cells were seeded at a density of 10^4^ cells per well in 96-well microtiter plates, and they were incubated overnight at 37°C with 5% CO_2_ in a 5% CO_2_ humidified environment in order to allow the attachment of cells at the bottom of the plates. Then, the cells were exposed to increasing concentrations of compounds (100, 50, 25, 10 and 5 μg/mL) dissolved in dimethyl sulfoxide (DMSO) and further diluted in fresh culture medium. In this assay, the final concentration of DMSO in the culture medium was 0.5% used as negative control while doxorubicin hydrochloride (Pfizer, United States of America) was used as a positive control. The culture plates were incubated for 48 h at 37°C in a 5% CO_2_ humidified environment, after which the culture medium was discarded, and replaced by 200 µL of fresh culture medium with 30 µL of 3-(4,5-dimethylthiazol-2-yl)-2,5-diphenyl-2H-tetrazolium bromide (MTT) (5 mg/mL) dissolved in phosphate buffered saline (Mosmann, 1983). After 4 h of incubation, the medium was gently removed, and the formazan crystals were solubilized in 50 µL of DMSO. The absorbance was measured at 570 nm after shaking for 1 min on a microplate reader (Synergy Multi-Mode Reader, BioTek, Winooski, United States of America).

The cell viability rate was determined at each concentration of the compound as a percentage of cells treated with DMSO at 0.5% used as negative control. The 50% inhibitory concentrations (IC_50_) were determined by using the non-linear regression graphical analysis of cell viability rate against the logarithm (log10) of compound concentrations with the software GraphPad Prism 6.0 (GraphPad software Inc., United States of America). The selectivity index (Supporting Material) values were calculated for each compound by dividing the IC_50_ of non-cancerous cells by the IC_50_ of cancerous cells in the same units (Mfotie Njoya et al., 2018; Mfotie Njoya et al., 2020).

### 2.6 Caspase-3/-7 luminescence assay

The effect of the most bioactive compounds (**2**), (**6**) and (**9**) were used for the analysis of caspase-3 and caspase-7 activities on MCF-7 cells by using the Caspase-Glo^®^ 3/7 kit (Promega, Germany). In fact, MCF-7 cells were seeded at a density of 10^4^ cells per well on 96-well microtiter plates, and the plates were incubated overnight at 37 °C in a 5% CO_2_ humidified environment. Then, the cells were exposed to the active compounds at different concentrations (½×IC_50_, IC_50_ and 2×IC_50_) or DMSO (0.5%) used as negative control, and further incubated for 18 h at 37°C in a 5% CO_2_ humidified environment. Thereafter, 100 µL of Caspase-Glo^®^ 3/7 reagent was added to each well, mixed and incubated in the dark for 1 h at room temperature. The luminescence was then measured on a microplate reader (Synergy Multi-Mode Reader, BioTek, Winooski, United States of America). The caspase-3/-7 activity was expressed as fold change of cells treated with 0.5% DMSO (control).

### 2.7 Anti-inflammatory assays

#### 2.7.1 Nitric oxide production inhibitory assay

Nitric oxide (NO) production was evaluated in lipopolysaccharide (LPS)-stimulated RAW 264.7 cells by measuring the influence of tested compounds on the accumulation of nitrite, an indicator of NO in the cell supernatant, which can be detected with Griess reagent (Merck, Darmstadt, Germany). Briefly, the RAW 264.7 cells at their exponential growth phase were seeded at a density of 2×10^4^ cells per well in 96 well-microtiter plates, and they were incubated overnight at 37°C in a 5% CO_2_ humidified environment to allow attachment. The cells were pre-treated with tested compounds (100 μg/mL) or DMSO 0.5% (negative control) and incubated for 1 h at 37°C in a 5% CO_2_ humidified environment. Then, culture medium containing LPS (2 μg/mL) was added to each well and further incubated for 24 h at 37°C in a 5% CO_2_ humidified environment. Thereafter, 100 μL of cell supernatant from each well were transferred into a new 96-well microtiter plate and an equal volume of Griess reagent was added according to protocol described by the manufacturer. The microtiter plate was incubated for 10 min in the dark at room temperature, and the absorbance of the mixture was measured at 550 nm on a microplate reader (Synergy Multi-Mode Reader, BioTek, Winooski, United States of America). The quantity of nitrite was determined from a sodium nitrite standard curve, and the percentage of NO production was calculated based on the ability of each tested sample to inhibit nitric oxide production by LPS-stimulated RAW 264.7 cells compared to the control (cells treated with LPS without samples which was considered as 100% NO production). Additionally, the cell viability of treated cells was determined by using the MTT assay as previously described (Mosmann, 1983). The whole experiment was repeated at different concentrations (100, 50, 25, 10 and 5 μg/mL) only for tested compounds that were able to inhibit at 50% of NO production, and the IC_50_ values was calculated as previously described (Mfotie Njoya et al., 2023).

#### 2.7.2 Soybean 15-LOX inhibitory assay

This assay is based on the formation of the Fe^3+^/xylenol orange (FOX) complex with maximal absorption at 560 nm (Pinto et al., 2007). Forty microliters of 15-LOX (final concentration: 200 UI/mL) from *Glycine* max (Merck, Darmstadt, Germany) was incubated with 20 µL of tested samples (100, 50, 25, 10, and 5 μg/mL) at 25°C for 5 min, with DMSO at 10% (*v*/*v*) being used as negative control. Thereafter, 40 µL of linoleic acid (final concentration, 140 μM) prepared in Tris-HCl buffer (50 mM, pH 7.4) was added, and the plates were further incubated at 25°C for 20 min in the dark. The assay was terminated by adding 100 μL of FOX reagent [sulfuric acid (30 mM), xylenol orange (100 μM), ferrous II) sulfate (100 μM), dissolved in methanol/water (9:1)], followed by the incubation of the plates at 25°C for 30 min in the dark. Finally, the absorbance was measured at 560 nm on a microplate reader (Epoch, BioTek, Winooski, United States of America). The blanks were made in the same way as tested samples except that the substrate was added after the FOX reagent. The 15-LOX inhibitory activity was calculated by using the following formula:

$$15-LOX\;inhibitory\;activity\;\left(\%\right)=100-\left[\frac{Abs\;\left(sample\right)-Abs\;\left(blank\right)}{Abs\;\left(negative\;control\right)-Abs\;\left(blank\right)}\times 100\right]$$ (1)

Abs: absorbance.

### 2.8 Statistical analysis

All experiments were performed in triplicate, and the results are presented as mean ± standard deviation (SD) values. The statistical analysis was done with the software GraphPad Prism 6.0 (GraphPad software Inc., United States of America) on which one-way analysis of variance (ANOVA) and Student–Newman–Keuls or Dunnett’s tests were used for the comparison of data among tested samples and/or controls. Results were considered significantly different when the *p*-value was greater than 0.05.

### 2.9 Molecular docking study

The X-ray crystal structures of putative target proteins in complex with inhibitors were retrieved from Protein Data Bank (https://www.rcsb.org/, accessed on 14 March 2023): Cyclooxygenase-2: COX-2 (PDB ID: 5KIR); Nuclear factor NF-kappa-B (p65 subunit): NFKB-p65 (PDB ID: 2RAM); Janus kinase 2: JAK2 (PDB ID: 6BBV); Signal transducer and activator of transcription 3: STAT3 (PDB ID: 6TLC); CASPASE-3 (PDB ID: 3DEI), CASPASE-7 (PDB ID: 1SHJ). PyMol v 2.0.7, was used to prepare the proteins for simulation by removing the inhibitors, water molecules, multi-chains and heteroatoms. MarvinSketch was used to draw the chemical structures of the ligands, and the Merck Molecular Force Field (MMFF94) as provided by MarvinSketch was used for energy minimization on the ligands (compounds) (Novichikhina et al., 2020). The energy minimization was carried out to make the ligands more stable near their initial states during molecular docking process. AutoDock Vina v 1.5.6 was used to add hydrogen atoms and Gasteiger charges accordingly to the ligands prior to molecular docking (Trott and Olson, 2010). The active sites of the target proteins were identified using CASTp 2.0 web-based tool (Tian et al., 2018). Further, AutoDock Vina v 1.5.6 was used to create a grid box around the identified active sites of the target proteins. Thereafter, standard precision ligand docking was performed using the same software. The best of nine binding poses based on the docking scores were visualized and captured in BIOVIA Discovery Studio Visualizer 2021 (Mfotie Njoya et al., 2023).

## 3 Results and discussion

### 3.1 Cytotoxic effect of isolated compounds and selectivity towards non-cancerous cells

**FIGURE 2:**
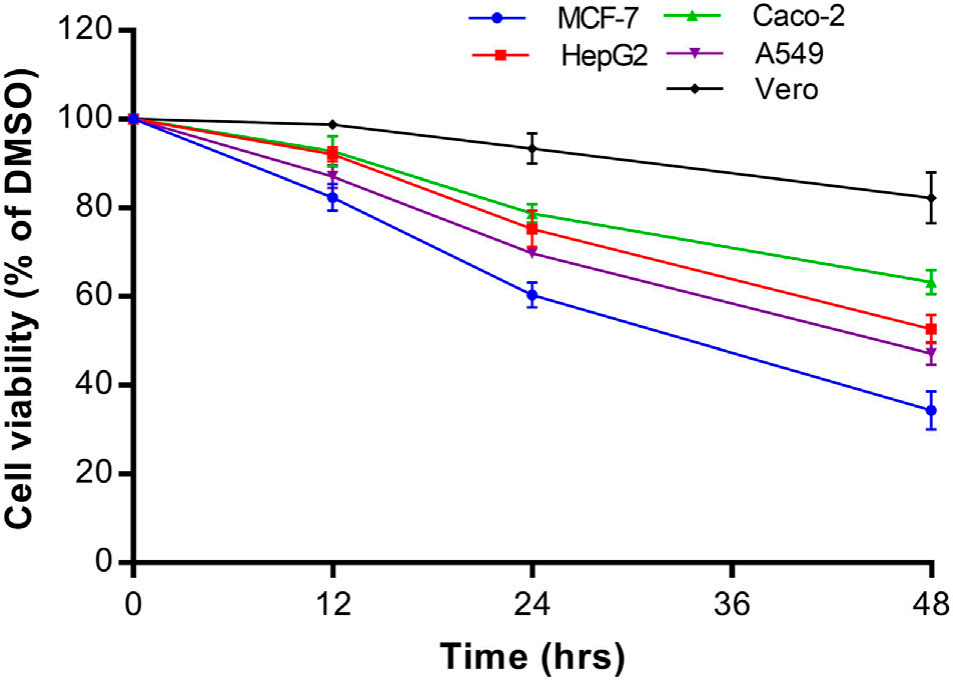
Time-dependent cytotoxic effect of hederagenin (**2**) at 10 μg/mL on different cancer cells (MCF-7, HepG2, Caco-2 and A549) and non-cancerous Vero cells. Data are presented as mean ± standard deviation of three independent experiments.

The growth inhibitory effect of natural compounds isolated from leaf, fruits and bark of *Sarcocephalus pobeguinii* (Rubiaceae) against four cancerous cells (MCF-7, HepG2, Caco-2 and A549) and non-cancerous Vero cells was expressed as percentage of respective cells treated with DMSO (0.5%) used as negative controls. Based on the results obtained for each compound tested at different concentrations, the 50% inhibitory concentrations (IC_50_) were determined and presented in Table 1. It was observed that hederagenin (**2**), quinovic acid 3-O-[α-D-quinovopyranoside] (**6**) and quinovic acid 3-O-[β-D-quinovopyranoside] (**9**) exhibited significant (*p* < 0.05) antiproliferative effect against all cancerous cells, especially for (**6**) which showed the highest cytotoxic effect on A549 cells with IC_50_ of 1.96 μg/mL (3.08 µM). Additionally, (**6**) was also toxic to the non-cancerous Vero cells with IC_50_ of 6.21 μg/mL (9.81 µM). By calculating the selectivity indexes (SI) as presented in Table 2, we found that (**6**), isolated from leaves of *S. pobeguinii,* had a poor selectivity (SI ≤ 1) (except for A549 cells) towards non-cancerous Vero cells where SI was 3.17. Similarly, (**9**), isolated from bark of *S. pobeguinii,* also had poor selectivity (SI ≤ 1) for all cancer types. On the other hand, (**2**), isolated from fruits of *S. pobeguinii*, was the only compound which had acceptable selectivity (1.24 ≤ SI ≤ 5.11) for all cancer cells towards non-cancerous Vero cells thereby suggesting that this compound can selectively kill cancer cells at the IC_50_ without causing harmful effects on non-cancerous cells. Therefore, regarding the fact that hederagenin (**2**) exhibited good selectivity, this compound was tested at 10 μg/mL on all cancer cells and non-cancerous cells for 12, 24 and 48 h in order to evaluate the time-dependent cytotoxic effect on cell growth. It was observed that (**2**) induced the growth inhibition of different cancer cells in a time-dependent manner, and the cytotoxic effect was more pronounced on cancer cells compared to non-cancerous cells (Figure 2). In fact, the MCF-7 cells followed by A549 cells were the most sensitive to the cytotoxic effect of hederagenin (**2**) which was less toxic to non-cancerous Vero cells. Interestingly, hederagenin, isolated from several plants, has been previously reported to potentially inhibit the proliferation of human lung cancer cells A549 (IC_50_ values of 26.3 and 39 μM) (Gauthier et al., 2009; Gao et al., 2016). In the current study, hederagenin inhibits A549 growth with an IC_50_ of 40.11 µM which is comparable to results obtained from previous works. Additionally, hederagenin was found to be less cytotoxic to human normal skin fibroblasts (WS1) cell lines (IC_50_ of 77 μM) (Gauthier et al., 2009) which also confirms the fact that this compound selectively kills cancer cells with least toxic effect to normal cells. Further, hederagenin, extracted in high quantities from the fruits of *Sapindus saponaria* L., (Sapindaceae), has been reported to be cytotoxic to different cancer cells, and structural modifications resulted to the development of potent anti-tumour compounds (Rodriguez-Hernandez et al., 2015; Rodriguez-Hernandez et al., 2016). Overall, hederagenin was isolated for the first time from fruits of *S. pobeguinii* (Rubiaceae), and our data corroborates its use as a potential candidate for the development of anticancer agents.

**TABLE 1:** Cytotoxic effects (IC_50_ values) of natural compounds isolated from roots, fruits, bark and leaves of *Sarcocephalus pobeguinii* and reference drug (doxorubicin) against cancer cell lines and non-cancerous Vero cells.

IC_50_ (µg/mL/µM)										
Compounds	Vero		MCF-7		HepG2		Caco-2		A549	
	µg/mL	µM	µg/mL	µM	µg/mL	µM	µg/mL	µM	µg/mL	µM
**1**	85.74 ± 1.64^a^	126.68 ± 2.42^a^	77.96 ± 2.79^a^	115.19 ± 4.51^a^	63.00 ± 1.29^a^	93.08 ± 1.91^a^	>100	>147.75	56.40 ± 3.79^a^	83.33 ± 5.07^a^
**2**	45.54 ± 2.34^b^	96.34 ± 4.95^b^	**8.92 ± 1.12** ^b^	**18.87 ± 2.37** ^b^	**24.88 ± 1.75** ^b^	**52.63 ± 3.70** ^b^	**36.69 ± 2.19** ^**a**^	**77.61 ± 4.63** ^**a**^	**18.96 ± 0.96** ^b^	**40.11 ± 2.03** ^**b**^
**3**	>100	>187.02	>100	>187.02	80.02 ± 1.71^c^	149.66 ± 3.20^c^	>100	>187.02	>100	>187.02
**4**	>100	>234.34	81.35 ± 1.48^a^	190.64 ± 3.47^c^	54.20 ± 1.64^d^	127.01 ± 3.84^d^	>100	>234.34	75.63 ± 3.74^c^	177.23 ± 5.16^c^
**5**	>100	>234.34	82.80 ± 1.35^a^	194.03 ± 3.16^c^	84.53 ± 1.45^c^	198.08 ± 3.40^e^	>100	>234.34	92.70 ± 3.73^d^	217.23 ± 5.77^d^
**6**	**6.21 ± 0.56** ^c^	**9.81 ± 0.79** ^c^	**5.96 ± 1.53** ^c^	**9.42 ± 2.41** ^d^	**41.94 ± 1.71** ^e^	**66.27 ± 2.70** ^f^	**7.41 ± 1.68** ^b^	**11.71 ± 2.65** ^b^	**1.96 ± 0.21** ^e^	**3.08 ± 0.33** ^**e**^
**7**	84.02 ± 3.27^a^	189.77 ± 5.68^d^	>100	>225.87	73.94 ± 4.22^c^	167.01 ± 6.05^c^	>100	>225.87	>100	>225.87
**8**	>100	>205.47	97.96 ± 5.72^a^	201.28 ± 8.02^c^	100.00 ± 3.82^f^	205.47 ± 5.84^e^	81.60 ± 3.88^c^	167.66 ± 5.97^c^	99.46 ± 3.85^d^	204.36 ± 5.01^d^
**9**	47.07 ± 3.66^b^	74.38 ± 5.78^e^	51.50 ± 4.93^d^	81.38 ± 5.96^d^	**47.22 ± 4.09** ^**e**^	**74.62 ± 6.62** ^**f**^	53.67 ± 4.97^d^	84.81 ± 5.85^a^	**44.22 ± 2.04** ^**f**^	**69.87 ± 3.96** ^**e**^
**10**	65.81 ± 2.29^d^	213.41 ± 4.12^d^	32.88 ± 3.78^e^	106.62 ± 2.25^a^	54.55 ± 3.51^d^	176.89 ± 4.35^c^	63.24 ± 4.04^d^	205.07 ± 3.10^d^	44.23 ± 4.59^a^	143.43 ± 6.61^f^
Doxorubicin (µM)	4.85 ± 0.51^f^		1.09 ± 0.06^e^		0.56 ± 0.05^g^		4.97 ± 0.74^e^		0.71 ± 0.05^g^	

Data are presented as means of triplicate measurements ± standard deviation. Superscript letters a–g represent statistical difference between data obtained, and for each column of the above table, data with same letters are statistically not different while data with different letters are significantly different at *p* < 0.05. IC50: concentration which inhibit 50% of cell growth compared to cells treated with DMSO (0.5%) used as negative control. Mixture of nauclealatifoline G and naucleofficine D (**1**), hederagenin (**2**) and chletric acid (**3**) were isolated from CH2Cl2/MeOH (1:2) fruit extract of S. pobeguinii. Taraxerol (**4**), α-amyrin(3β-hydroxy-urs-12-en-3-ol) (**5**) and quinovic acid 3-O-[α-D-quinovopyranoside] (**6**) were isolated from methanol leaf extract of S. pobeguinii. Erythrodiol (**7**), quinovic acid (**8**), quinovic acid 3-O-[β-D-quinovopyranoside] (**9**) and latifoliamide C (**10**) were isolated from the methanol bark extract of S. pobeguinii.

Bold values mean compounds significantly active in the assay.

**TABLE 2:** Selectivity index (SI) values of natural compounds.

Compounds	SI values			
	MCF7	HepG2	Caco-2	A549
**1**	1.10	1.36	nd	**1.52**
**2**	**5.11**	**1.83**	1.24	**2.40**
**3**	nd	nd	nd	nd
**4**	nd	nd	nd	nd
**5**	nd	nd	nd	nd
**6**	1.04	0.14	0.83	**3.17**
**7**	nd	1.13	nd	nd
**8**	nd	nd	nd	nd
**9**	0.91	0.99	0.88	1.06
**10**	**2.01**	1.20	1.04	**1.49**
Doxorubicin	**4.45**	**8.66**	0.98	**6.83**

Mixture of nauclealatifoline G and naucleofficine D (**1**), hederagenin (**2**) and chletric acid (**3**) were isolated from CH2Cl2/MeOH (1:2) fruit extract of S. pobeguinii. Taraxerol (**4**), α-amyrin(3β-hydroxy-urs-12-en-3-ol) (**5**) and quinovic acid 3-O-[α-D-quinovopyranoside] (**6**) were isolated from methanol leaf extract of S. pobeguinii. Erythrodiol (**7**), quinovic acid (**8**), quinovic acid 3-O-[β-D-quinovopyranoside] (**9**) and latifoliamide C (**10**) were isolated from the methanol bark extract of S. pobeguinii. nd: not determined.

Bold values mean compounds with good selectivity index.

### 3.2 Structure–activity relationship

Three triterpenoids including two saponins isolated from *S. pobeguinii* were cytotoxic to cancer cells, and their efficacy varied according to their structural configuration or the presence of some functional groups. When compared to other triterpenoids, the cytotoxic effect of the two bioactive saponins, namely, quinovic acid 3-O-[α-D-quinovopyranoside] (**6**) and quinovic acid 3-O-[β-D-quinovopyranoside] (**9**) can be attributed to the presence of a pyranose moiety attached at position C-3. Moreover, the α anomer (**6**) is highly cytotoxic compared to the β anomer (**9**) which suggests that the configuration of the glycosidic bond (C-O-sugar bond) has an impact on the cytotoxic effect. According to several reports, the anticancer activity of triterpenoid saponins is strongly linked to the presence of functional carboxylic and hydroxyl groups on the aglycone chain, the stereo-selectivity and the type of sugar molecule attached (Nag et al., 2012; Xu et al., 2013; Elekofehinti et al., 2021; Podolak et al., 2023). In contrast, we observed from our study that despite the presence carboxylic and hydroxyl groups, other triterpenoids (**3**), (**4**), (**5**), (**7**) and (**8**) were less cytotoxic or inactive compared to hederagenin (**2**) which strongly inhibit the growth of several cancer cells. We therefore suggest that the efficacy of hederagenin might be attributed to the asymmetric carbon at position C-4 which yield a different conformation due to irregular spatial arrangements of chemical groups. This conformation might ease the interaction with molecular targets involved in the activation of cancer cell death pathways. Further, the solubility of hederagenin in culture media may also be responsible for its efficacy on cancer cells.

### 3.3 Caspase-dependent activation by bioactive compounds

**FIGURE 3:**
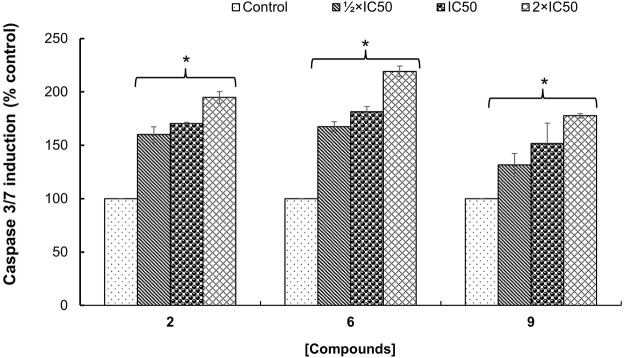
Fold change in the enzymatic activity of caspase-3/-7 expressed as percentage of control (cells treated with DMSO 0.5%) on MCF-7 cells treated with hederagenin (2), quinovic acid 3-O-[α-D-quinovopyranoside] (6) and quinovic acid 3-O-[β-D-quinovopyranoside] (9), respectively. Data are presented as mean ± SD of three independent experiments. *p < 0.05 indicate the significant difference compared to the control using the Dunnett’s test.

The potential mechanism of action of bioactive compounds on cellular viability was explored by quantifying the caspase-3/-7 activity in MCF-7 cells. It was found that the caspase-3/-7 activity was significantly (*p* < 0.05) induced in a concentration-dependent manner compared to control cells treated with DMSO 0.5% (Figure 3). Additionally, the optimal effect was observed with hederagenin (**2**) and quinovic acid 3-O-[α-D-quinovopyranoside] (**6**) tested at 2×IC_50_ where an induction up to 2-fold change in the activation of caspase-3/-7 activity was recorded. Caspases 3 and 7 are known as “executioners” of apoptosis, and the combined role of both caspases is crucial for the activation of apoptotic pathways (Olsson and Zhivotovsky, 2011). In fact, caspase 3 controls DNA fragmentation and morphologic changes of apoptosis, while caspase 7 is more important for the loss of cellular viability (Lakhani et al., 2006). Therefore, the induction of caspase-3/-7 activity after treatment with bioactive compounds implied that the activation of apoptotic pathways is involved in the mechanism of induced cell death in MCF-7 cells.

### 3.4 Anti-inflammatory effect of isolated compounds

**FIGURE 4:**
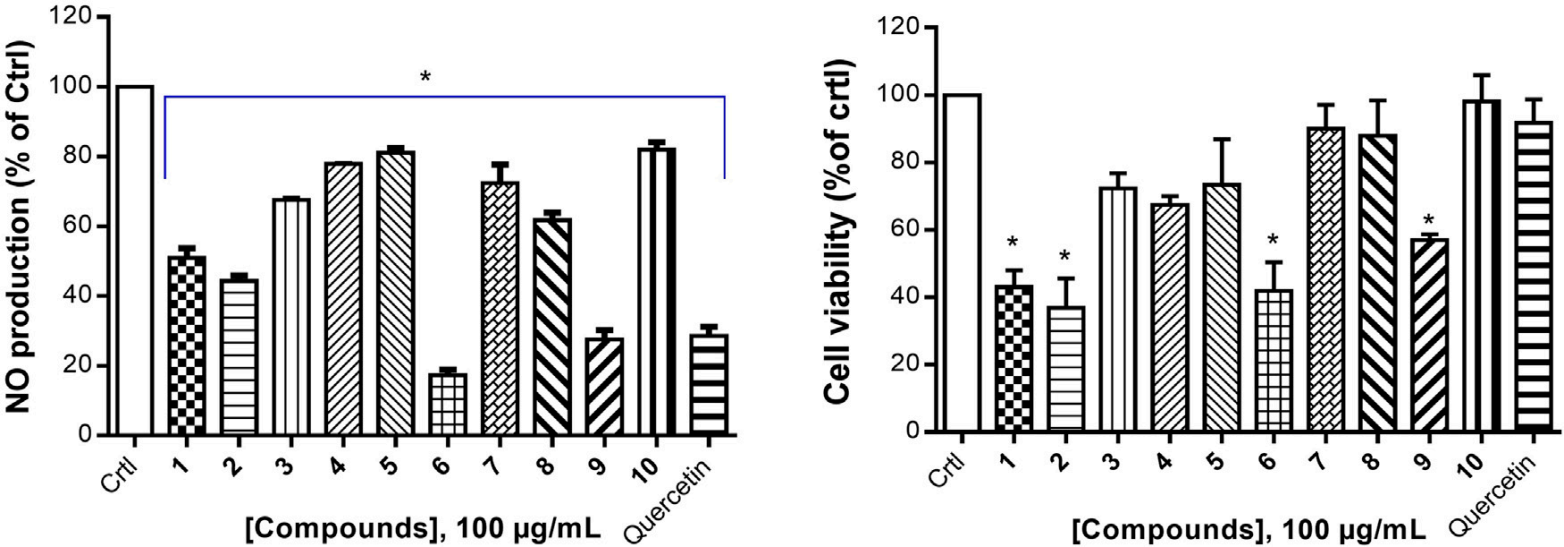
Nitric oxide production inhibition by natural compounds in LPS-stimulated RAW 264.7 cells **(A)** and their respective percentage of cell viability **(B)**. The NO production inhibition was determined based on the efficacy of tested samples to reduce NO release by LPS- stimulated RAW 264.7 cells compared with the negative control (RAW 264.7 cells treated with LPS and DMSO at 0.5% without test samples), which was considered to be 100% of NO production. Quercetin was used as a positive control. Data represent the mean ± standard deviation of three independent experiments; (*) means a statistical difference (*p* < 0.05) between the tested samples *versus* the negative control (Crtl).

Cancer development is known as a multistep process during which inflammation is one of the factors contributing to the promotion of cell proliferation, invasion, angiogenesis, and metastasis. Moreover, it has been proven that patients on Nonsteroidal anti-inflammatory drugs (NSAIDs) are at reduced risk of cancer development (Singh et al., 2019). One of the possible reasons is that anti-inflammatory drugs might also interfere with signaling pathways modulated in both inflammation and cancer. Therefore, we decided to investigate the anti-inflammatory effect of compounds isolated from *S. pobeguinii* on two experimental models. We found that all compounds (tested at 100 μg/mL) inhibited the NO production in LPS-stimulated RAW 264.7 cells, with the mixture nauclealatifoline G and naucleofficine D (**1**), hederagenin (**2**), quinovic acid 3-O-[α-D-quinovopyranoside] (**6**) and quinovic acid 3-O-[β-D-quinovopyranoside] (**9**) being the most active by reducing at least 50% of NO production (Figure 4A). However, the NO production inhibitory effect of compounds (**1**), (**2**), (**6**) and (**9**) might be attributed to their cytotoxic effect on LPS-stimulated RAW 264.7 cells as it was observed that these compounds significantly (*p* < 0.05) reduced the cell viability (Figure 4B). Besides, by determining the IC_50_ values as presented in Table 3, we found that (**6**) and (**9**) strongly inhibits NO production in a concentration-dependent manner with IC_50_ values of 28.71 and 77.36 µM, respectively. On the other hand, we examined the inhibitory effect of isolated compounds on the activity of 15-LOX, an enzyme which regulate the inflammatory responses via the generation of pro-inflammatory mediators known as leukotrienes. It resulted that the mixture of nauclealatifoline G and naucleofficine D (**1**), hederagenin (**2**) and chletric acid (**3**) strongly inhibited the activity of 15-LOX with IC_50_ values of 16.51, 28.20 and 41.17 µM, respectively (see Table 3). These compounds were even more active than quercetin, which had an IC_50_ value of 61.94 µM. Based on the fact that 15-LOX and NO are among the mediators involved in the progression of various inflammation-related diseases including cancer (Zhao et al., 2021), our study therefore identified compounds which can be considered as potential anticancer agents with additional anti-inflammatory effect for the effective management of both diseases.

**TABLE 3:** Anti-inflammatory effect (IC_50_ values) of natural compounds isolated from fruits, leaves and bark of *Sarcocephalus pobeguinii*.

IC_50_ values				
Compounds	NO		15-LOX	
	µg/mL	µM	µg/mL	µM
**1**	94.92 ± 4.66^a^	140.25 ± 5.89^a^	**11.17 ± 0.96** ^a^	**16.51 ± 1.42** ^a^
**2**	65.13 ± 1.23^b^	137.78 ± 2.17^a^	**13.43 ± 1.59** ^a^	**28.20 ± 3.71** ^b^
**3**	>100	>187.02	**22.01 ± 1.39** ^b^	**41.17 ± 2.61** ^**c**^
**4**	>100	>234.34	>100	>234.34
**5**	>100	>234.34	>100	>234.34
**6**	**18.17 ± 1.26** ^c^	**28.71 ± 1.99** ^b^	>100	>158.02
**7**	95.63 ± 4.95^a^	216.00 ± 6.18^c^	65.23 ± 4.45^c^	147.33 ± 6.07^d^
**8**	86.02 ± 2.09^d^	176.74 ± 4.29^d^	**34.97 ± 2.68** ^**d**^	**71.86 ± 4.67** ^**e**^
**9**	**48.96 ± 4.92** ^**e**^	**77.36 ± 5.77** ^**e**^	74.81 ± 2.19^e^	118.22 ± 3.46^f^
**10**	>100	>324.27	78.09 ± 4.13^e^	253.22 ± 7.40^g^
Quercetin	**12.85 ± 1.59** ^**f**^	**42.52 ± 4.99** ^**f**^	**18.72 ± 2.72** ^**b**^	**61.94 ± 4.51** ^**h**^

Data are presented as means of triplicate measurements ±standard deviation. Superscript letters a–h represent statistical difference between data obtained, and for each column of the above table, data with same letters are statistically not different while data with different letters are significantly different at *p* < 0.05. IC50: concentration of the tested samples which inhibit 50% of biological effect. Mixture of nauclealatifoline G and naucleofficine D (**1**), hederagenin (**2**) and chletric acid (**3**) were isolated from CH2Cl2/MeOH (1:2) fruit extract of S. pobeguinii. Taraxerol (**4**), α-amyrin(3β-hydroxy-urs-12-en-3-ol) (**5**) and quinovic acid 3-O-[α-D-quinovopyranoside] (**6**) were isolated from methanol leaf extract of S. pobeguinii. Erythrodiol (**7**), quinovic acid (**8**), quinovic acid 3-O-[β-D-quinovopyranoside] (**9**) and latifoliamide C (**10**) were isolated from the methanol bark extract of S. pobeguinii.

Bold values mean compounds significantly active in the assay.

### 3.5 Binding efficacy of bioactive compounds against putative molecular targets

**FIGURE 5:**
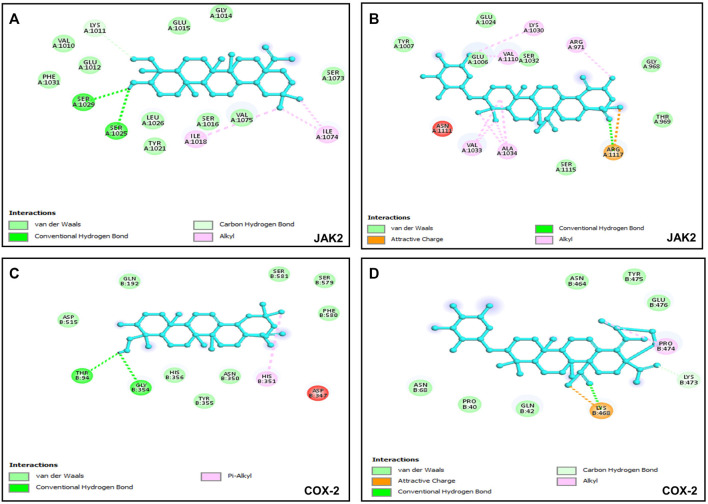
Visualization of binding interactions by using BIOVIA Discovery Studio Visualizer 2021 software; **(A)** protein-ligand interactions between JAK2 and hederagenin (−6.8 kcal/mol); **(B)** protein-ligand interactions between JAK2 and quinovic acid 3-O-[*α*-D-quinovopyranoside] (−6.4 kcal/mol); **(C)** protein-ligand interactions between COX-2 and hederagenin (−6.1 kcal/mol); **(D)** protein-ligand interactions between COX-2 and quinovic acid 3-O-[*α*-D-quinovopyranoside] (−6.7 kcal/mol).

Six putative proteins, namely, COX-2, NFκB-p65, JAK2, STAT3, CASPASE-3 and CASPASE-7 were chosen for molecular docking based on the fact that they are frequently used as therapeutic targets to regulate inflammatory signaling pathways in cancer treatment (Jha et al., 2022). As presented in Table 4, the docking score (kcal/mol) of the best binding pose was determined for each ligand against the selected proteins. According to these results, hederagenin (**2**), quinovic acid 3-O-[α-D-quinovopyranoside] (**6**) and quinovic acid 3-O-[β-D-quinovopyranoside] (**9**) exhibited activity within the range of −4.3 to −6.8 kcal/mol, where −6.8 kcal/mol was the most effective interaction between JAK2 and (**9**), and −4.3 kcal/mol was the lowest binding score between CASPASE-3 and (**2**). Taken individually, each of the three bioactive compounds exhibited the greatest binding efficacy (binding score above −6 kcal/mol) against JAK2 and COX-2 which suggests that these proteins are the potential molecular targets involved in their antiproliferative and anti-inflammatory effects (Figure 5). In addition, STAT3 and NFKB-p65 also interacted well with bioactive compounds. According to the literature reports, the high activation of JAK2/STAT3 signaling pathway, frequently detected in various tumors, has recently emerged as a new site for the development of novel anti-tumor agents, and these proteins (JAK2 and STAT3) are promising therapeutic targets for the treatment of many solid tumors (Huang et al., 2022; Mengie Ayele et al., 2022). Moreover, NFκB-p65 is constitutively activated in many human cancers, where it contributes to almost all steps of tumorigenesis including sustained proliferation, cell death resistance, tumor-promoting inflammation, tissue invasion, angiogenesis, and metastasis (Lin et al., 2010). As such, the NF-κB pathway is an attractive therapeutic target in a broad range of human cancers, as well as in numerous non-malignant diseases. Furthermore, COX-2, an enzyme that catalyzes the first step in the synthesis of prostanoids, is associated with inflammatory diseases and carcinogenesis (Liu et al., 2015). An overexpression of COX-2 is observed in cancers of the pancreas, breast, colorectal, stomach, and lung carcinoma. Therefore, COX-2 is considered as a significant target for the development of new anticancer agents (Mohsin et al., 2022). Among the bioactive compounds investigated, the mechanism of action of hederagenin has been extensively studied in several studies. In fact, hederagenin has been reported to have anti-tumour and anti-inflammatory effects by regulating the NFκB, PI3K/AKT and JAK2/STAT3/MAPK signalling pathways, and by reducing the expression of pro-inflammatory cytokines or enzyme production, including Tumor Necrosis Factor- α (TNF-α), interleukin 6 (IL-6), NO, prostaglandin E2 (PGE_2_), inducible nitric oxide synthase (iNOS) and COX-2 (Zhang et al., 2012; Lee et al., 2015; Kim et al., 2017; Yu et al., 2020; Shen et al., 2023). Our results indicate the accordance between the molecular docking and the previous studies thereby confirming the efficacy of hederagenin as a prominent lead compound for the management of inflammation and cancer development. Further investigations are required for quinovic acid 3-O-[α-D-quinovopyranoside] and quinovic acid 3-O-[β-D-quinovopyranoside] which modes of action are still not experimentally investigated.

**TABLE 4:** Molecular docking score (kcal/mol) and interacting residues of the most bioactive compounds against six putative target proteins based on the best protein-ligand binding pose.

Compounds	Putative targets	Docking score values (kcal/mol)	Interacting residues
Hederagenin	COX-2	−6.1	B chain: THR94, GLY354, HIS351
	NFκB-p65	−5.7	A chain: ARG236, SER240, PHE239
	STAT3	−5.3	A chain: VAL537,ILE522, ALA505, LEU525,TRP501
	JAK2	−6.8	A chain: SER1025, SER1029, ILE1018, SER1074
	CASPASE-3	−4.3	C chain: LEU168, PHE256, ALA254
	CASPASE-7	−4.4	A chain: ARG87, CYS186
Quinovic acid 3-O-[*α*-D-quinovopyranoside]	COX-2	−6.7	B chain: LYS468, LYS473, PRO474
	NFκB-p65	−5.2	B chain: ARG236, PRO256, PHE239
	STAT3	5.0	B chain: THR526, LUE525, ALA505
	JAK2	−6.4	A chain: LYS1030, ARG1034, ARG1117
	CASPASE-3	−4.9	C chain: LEU168, PHE246, ALA254
	CASPASE-7	−5.3	A chain: TRP 240, VAL220, HIS144
Quinovic acid 3-O-[β-D-quinovopyranoside]	COX-2	−6.5	B chain: GLN192, PHE580, SER581, SER579, VAL582
	NFκB-p65	−5.0	B chain: ASP53,LYS28, ARG30
	STAT3	5.8	B chain: SER399, LEU260, CYS259, PRO256, ILE258, ALA250, CYS257, ARG325, PRO366
	JAK2	−6.8	A chain: SER1029, SER1025, GLU1012, TYR1021, LEU1026, VAL1025, PRO1013, ILE1018, ILE1074, PHE1019
	CASPASE-3	−5.3	A chain: THR62, THR166, MET61, PHE128, GLY122, C chain: THR166, LEU168
	CASPASE-7	−5.7	A chain: ARG236, SER240, PHE239

## 4 Conclusion

This study reported the isolation of ten natural compounds from leaf, fruit and bark of *Sarcocephalus pobeguinii*, and described their antiproliferative and anti-inflammatory effects as well as exploration of potential target proteins of bioactive compounds. Hederagenin (**2**), quinovic acid 3-O-[α-D-quinovopyranoside] (**6**) and quinovic acid 3-O-[β-D-quinovopyranoside] (**9**) exhibited antiproliferative effect against all cancerous cells by inducing apoptosis via caspase-3/-7 activation. Among these bioactive compounds, hederagenin, isolated for the first time from the fruits of *S. pobeguinii*, selectively kills cancer cells with additional anti-inflammatory potential, and it interacts with JAK2 and COX-2 suggesting these proteins as its potential molecular targets. The molecular docking results were in agreement with previously reported experimental data thereby confirming hederagenin as a prominent drug candidate to tackle cancer progression. Furthermore, apart from hederagenin, nauclealatifoline G and naucleofficine D (**1**), and chletric acid (**3**) strongly inhibited the activity of 15-LOX as compared to quercetin, which opens further research direction aiming to investigate experimentally their mode of action on molecular targets of inflammation.

## Data Availability

The original contributions presented in the study are included in the article/Supplementary Material, further inquiries can be directed to the corresponding authors.
