# Supplementary material for: In vitro antiproliferative, anti-inflammatory effects and molecular docking studies of natural compounds isolated from Sarcocephalus pobeguinii (Hua ex Pobég)
Source: Front Pharmacol. 2023 Jun 21;14:1205414. doi: 10.3389/fphar.2023.1205414 (PMC10320002; doi:10.3389/fphar.2023.1205414)
Supplement: Supplementary file 1 [file DataSheet1.docx]

**Supplementary data**

***In vitro* Antiproliferative, Anti-inflammatory Effects** **and Molecular Docking Studies of Natural Compounds Isolated from *Sarcocephalus pobeguinii* (Hua ex Pobég).**

Emmanuel Mfotie Njoya ^1,2,^*, Brigitte Ndemangou^4^, Jude Akinyelu ^5^, Aristide M. Munvera ^3^, Chika. I. Chukwuma ^1^, Yves M. Nguekeu Mba ^1^, Pierre Mkounga ^3^, Samson S. Mashele ^1^, Tshepiso J. Makhafola ^1^*, and Lyndy J. McGaw ^2^

^1^ Centre for Quality of Health and Living, Faculty of Health and Environmental Sciences, Central University of Technology, Bloemfontein 9301, South Africa; [cchukwuma@cut.ac.za](mailto:cchukwuma@cut.ac.za) (C.I.C.); [smashele@cut.ac.za](mailto:smashele@cut.ac.za) (S.S.M.), [ynguekeu@cut.ac.za](mailto:ynguekeu@cut.ac.za) (Y.M.N.M.)

^2^ Department of Paraclinical Sciences, Faculty of Veterinary Science, University of Pretoria, Onderstepoort, Pretoria 0110, South Africa; [lyndy.mcgaw@up.ac.za](mailto:lyndy.mcgaw@up.ac.za) (L.J.M.)

^3^ Department of Organic Chemistry, Faculty of Science, University of Yaoundé I, Yaoundé **812**, Cameroon; [mfifena@hotmail.fr](mailto:mfifena@hotmail.fr) (A.M.M.); [mpierrendi@yahoo.fr](mailto:mpierrendi@yahoo.fr) (P.M.)

^4^ University Institute of Technology of Wood Technology, Mbalmayo 306, Cameroon; [ndemangou@yahoo.fr](mailto:ndemangou@yahoo.fr) (B.N.)

^5^ Department of Biochemistry, Federal University Oye-Ekiti, Ekiti State, Nigeria; [jude.akinyelu@fuoye.edu.ng](mailto:jude.akinyelu@fuoye.edu.ng) (J.A.)

***** Correspondence: [mfotiefr@yahoo.fr](mailto:mfotiefr@yahoo.fr) or [enjoya@cut.ac.za](mailto:enjoya@cut.ac.za) (E.M.N.); [jmakhafola@cut.ac.za](mailto:jmakhafola@cut.ac.za) (T.J.M.)

**Table of Contents:**

**Figure S1:** Mass spectrum of mixture Nauclealatifoline G and Naucleofficines D **(1).**

**Figure S2:** ^1^H NMR spectrum (DMSO-*d_6_*, 400 MHz) of mixture Nauclealatifoline G and Naucleofficines D (**1**).

**Figure S3:** ^13^C NMR spectrum (DMSO-*d_6_*, 100 MHz) of mixture Nauclealatifoline G and Naucleofficines D (**1**).

**Figure S4:** ^1^H NMR spectrum (DMSO-*d_6_*, 400 MHz) of hederagenin (**2**).

**Figure S5**: ^13^C NMR spectrum (DMSO-*d_6_*, 100 MHz) of hederagenin (**2**).

**Figure S6:** ^1^H NMR spectrum (MeOH-*d_4_*, 400 MHz) of chletric acid (**3**)

**Figure S7:** ^13^C NMR spectrum (MeOH-*d_4_*, 100 MHz) of chletric acid (**3**)

**Figure S8:** ^1^H NMR spectrum (pyridine-*_d5_*, 400 MHz) of taraxerol (**4**)

**Figure S9:** ^13^C NMR spectrum (pyridine-*_d5_*, 100 MHz) of taraxerol (**4**)

**Figure S10:** ^1^H NMR spectrum (CDCl_3_, 400 MHz) of *α*-amyrin (3β-hydroxy-urs-12-en-3-ol) (**5**)

**Figure S11:** ^13^C NMR spectrum (CDCl_3_, 100 MHz) of *α*-amyrin (3β-hydroxy-urs-12-en-3-ol) (**5**)

**Figure S12-1:** ^1^H NMR spectrum (DMSO-*d_6_*, 400 MHz) of quinovic acid 3-O-[α-D-quinovopyranoside] (**6**).

**Figure S12-2:** ^1^H NMR spectrum (DMSO-*d_6_*, 400 MHz) of quinovic acid 3-O-[α-D-quinovopyranoside] (**6**).

**Figure S13:** ^13^C NMR spectrum (DMSO-*d_6_* , 100 MHz) of quinovic acid 3-O-[α-D-quinovopyranoside] (**6**).

**Figure S14:** ^1^H NMR spectrum (CDCl_3_, 400 MHz) of erythrodiol (**7**).

**Figure S15:** ^13^C NMR spectrum (CDCl_3_, 100 MHz) of erythrodiol (**7**).

**Figure S16:** ^1^H NMR spectrum (DMSO-*d_6_*, 400 MHz) of quinovic acid (**8**).

**Figure S17:** ^13^C NMR spectrum (DMSO-*d_6_*, 100 MHz) of quinovic acid (**8**).

**Figure S18:** Mass spectrum of quinovic acid 3-O-[β-D-quinovopyranoside] (**9**).

**Figure S19:** ^1^H NMR spectrum (MeOH-*d_4_*, 500 MHz) of quinovic acid 3-O-[β-D-quinovopyranoside] (**9**).

**Figure S20:** ^13^C NMR spectrum (MeOH-*d_4,_* 125MHz) of quinovic acid 3-O-[β-D-quinovopyranoside] (**9**).

**Figure S21:** ^1^H NMR spectrum (CD3OD, 400 MHz) of latifoliamide C (**10**).

**Figure S22:** ^13^C NMR spectrum (CD3OD, 100 MHz) of latifoliamide C (**10**).

**
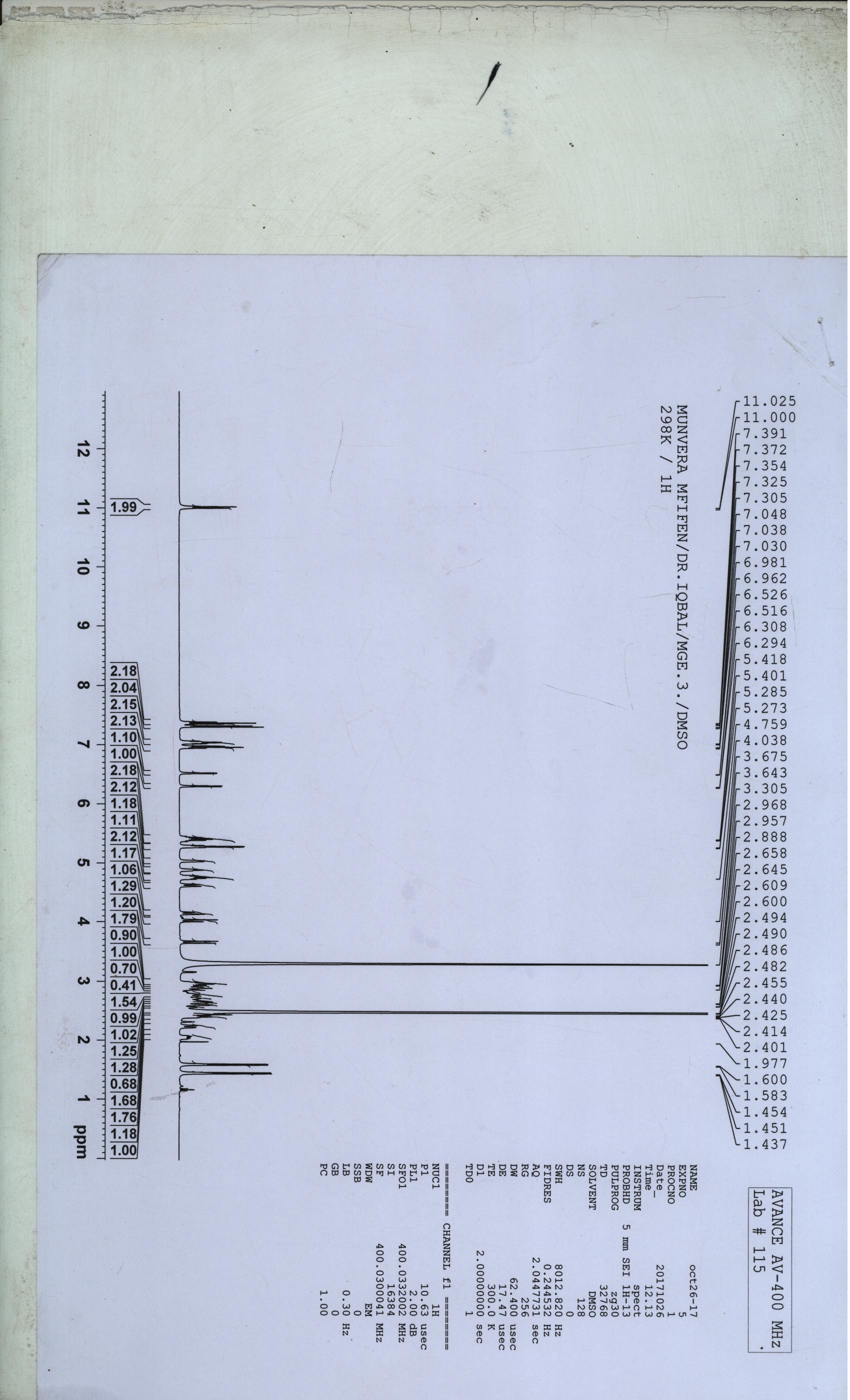
**
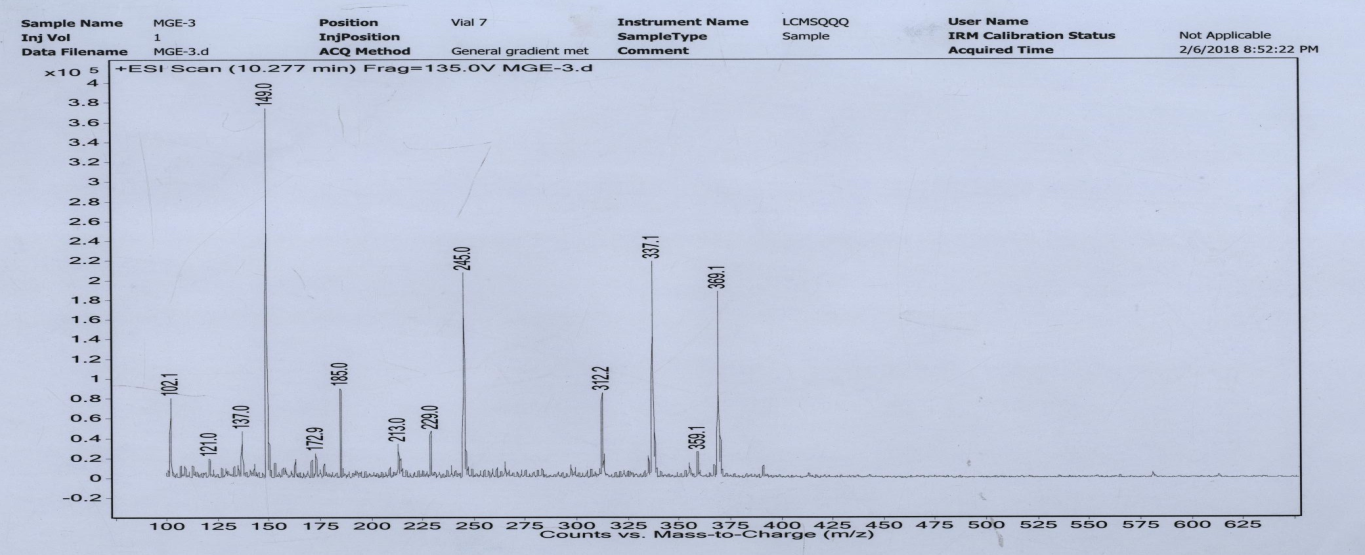


**Figure S1:** Mass spectrum of mixture Nauclealatifoline G and Naucleofficines D (**1**).

**Figure S2:** ^1^H NMR spectrum (DMSO-*d_6_*, 400 MHz) of mixture Nauclealatifoline G and Naucleofficines D (**1**).


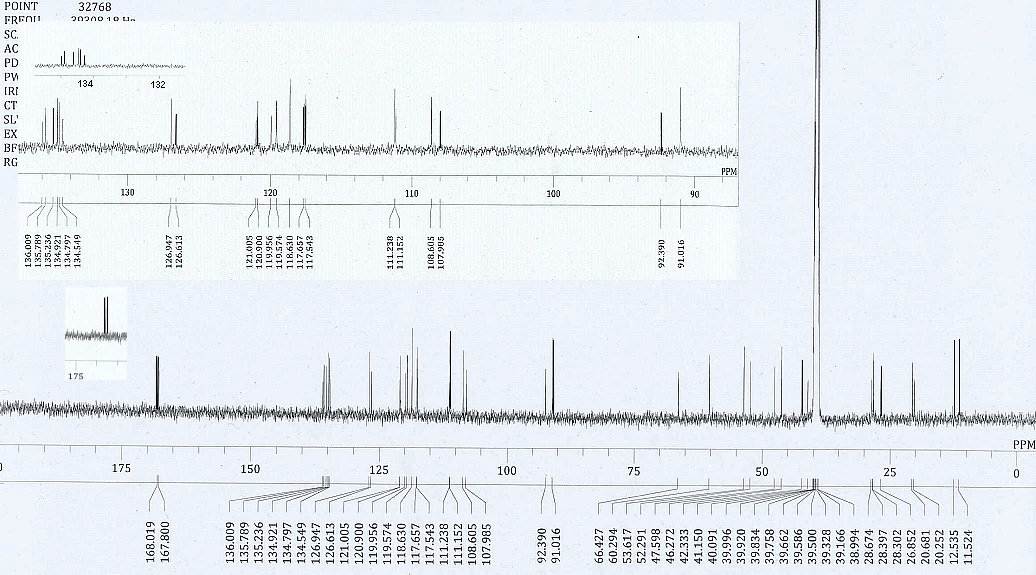


**Figure S3:** ^13^C NMR spectrum (DMSO-*d_6_*, 100 MHz) of mixture Nauclealatifoline G and Naucleofficines D (**1**).


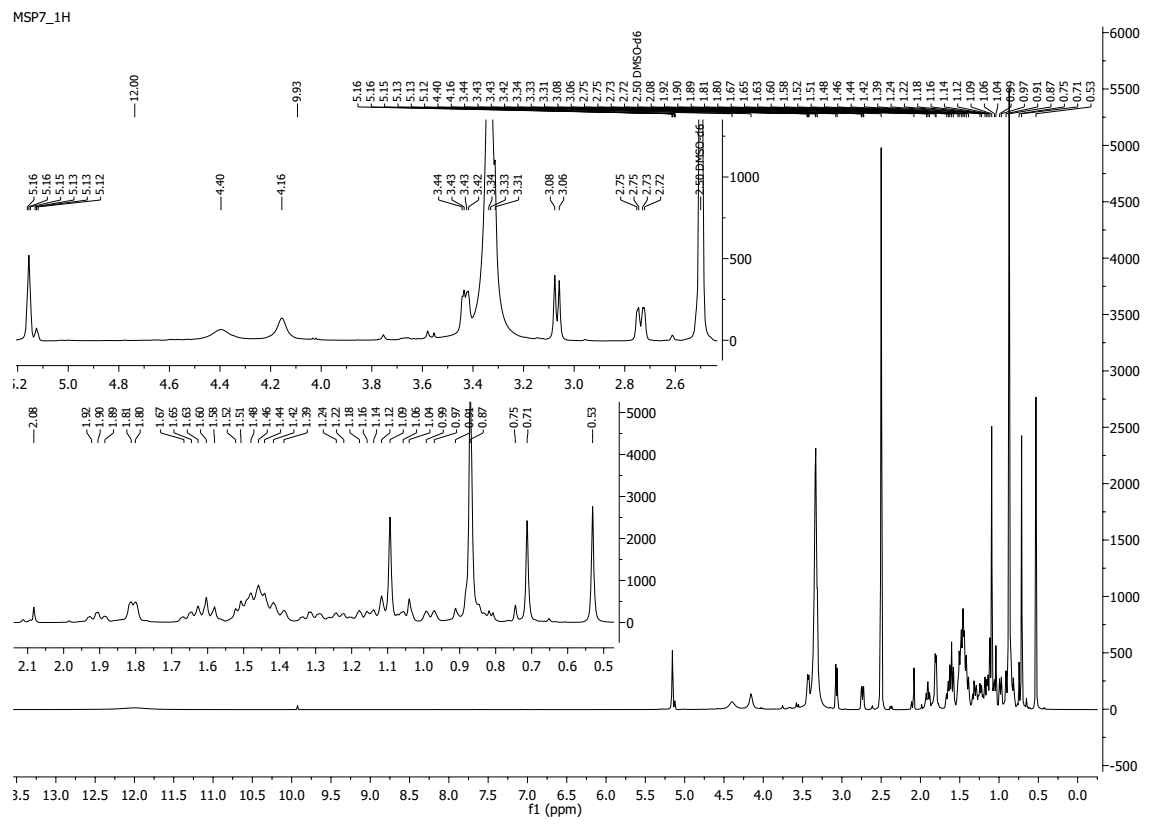


**Figure S4:** ^1^H NMR spectrum (DMSO-*d_6_*, 400 MHz) of hederagenin (**2**).


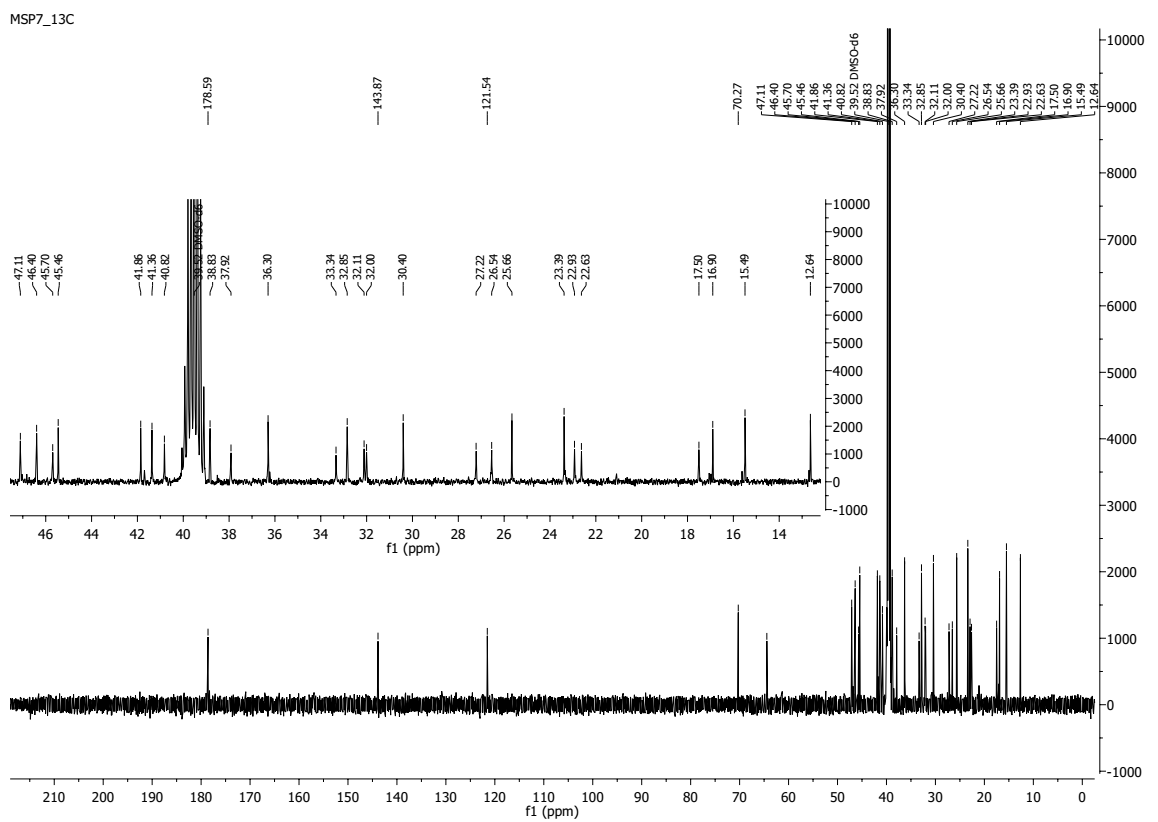


**Figure S5:** ^13^C NMR spectrum (DMSO-*d_6_*, 100 MHz) of hederagenin (**2**).


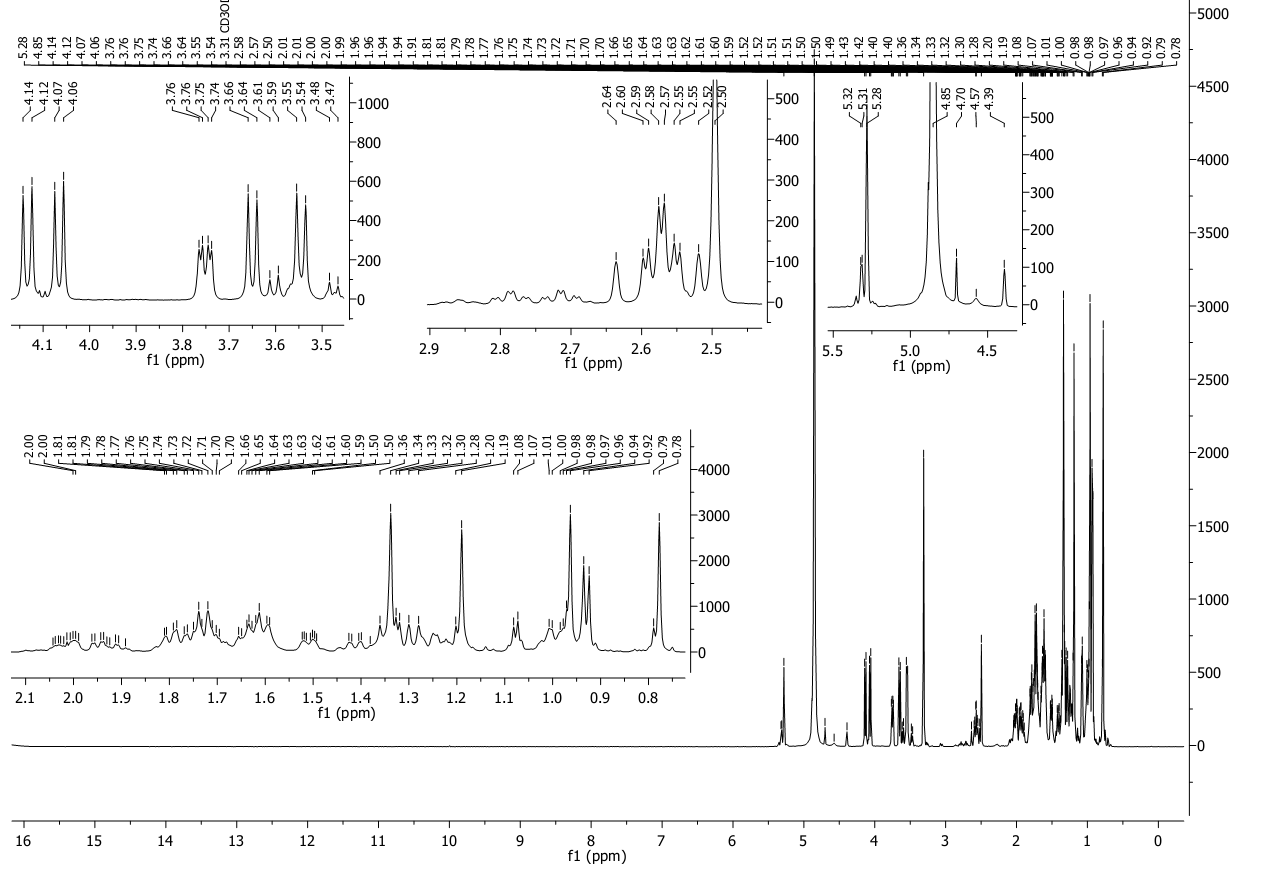


**Figure S6:** ^1^H NMR spectrum (MeOH-*d_4_*, 400 MHz) of chletric acid (**3**)


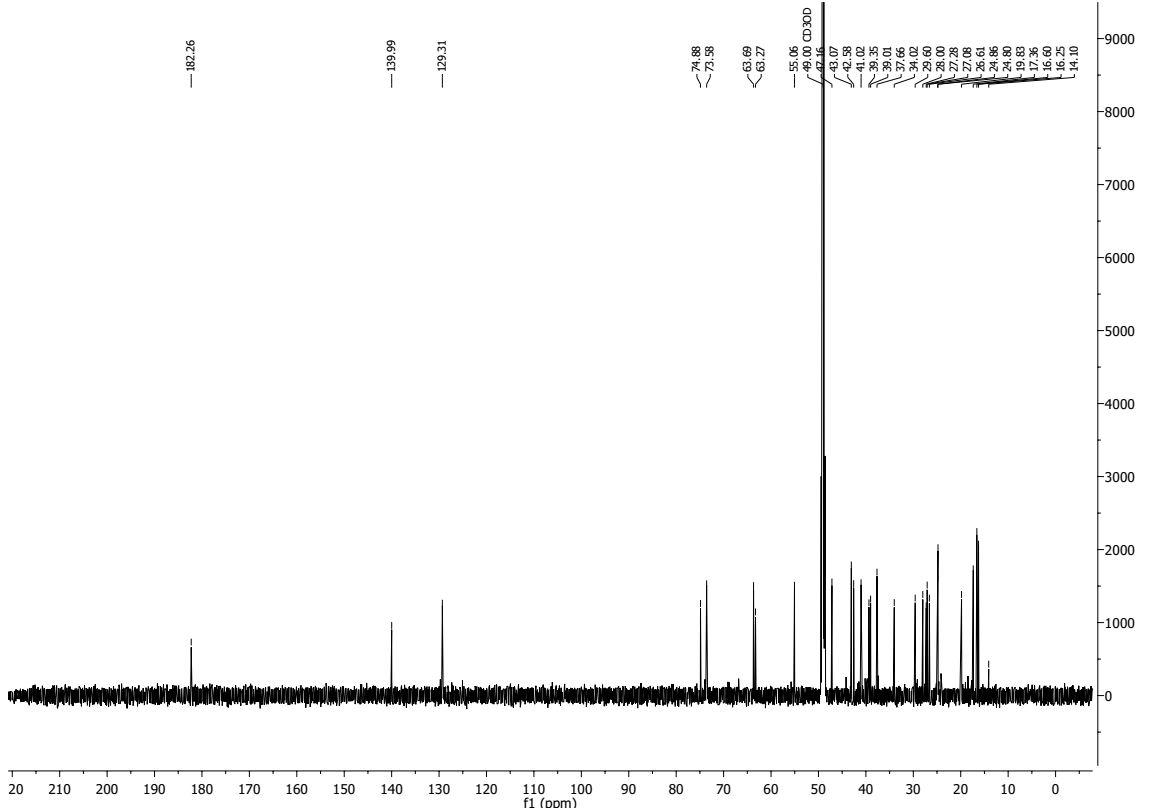


**Figure S7:** ^13^C NMR spectrum (MeOH-*d_4_*, 100 MHz) of chletric acid (**3**)


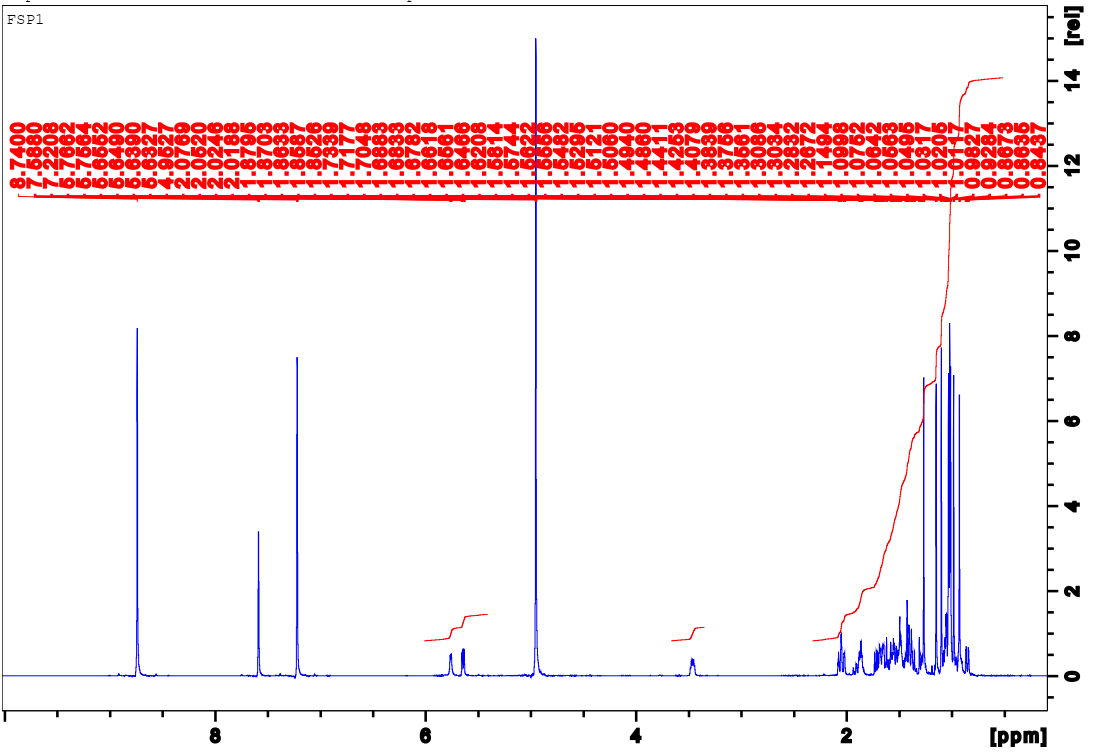


**Figure S8:** ^1^H NMR spectrum (pyridine-*_d5_*, 400 MHz) of taraxerol (**4**)


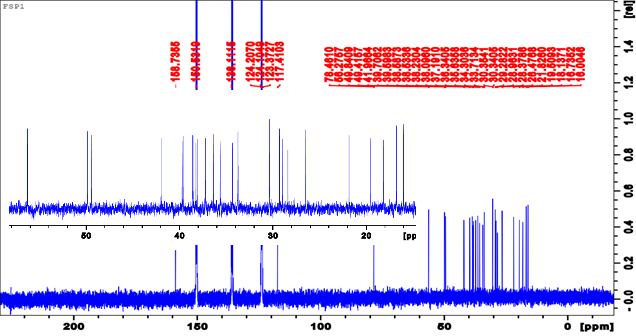


**Figure S9:** ^13^C NMR spectrum (pyridine-*_d5_*, 100 MHz) of taraxerol (**4**)


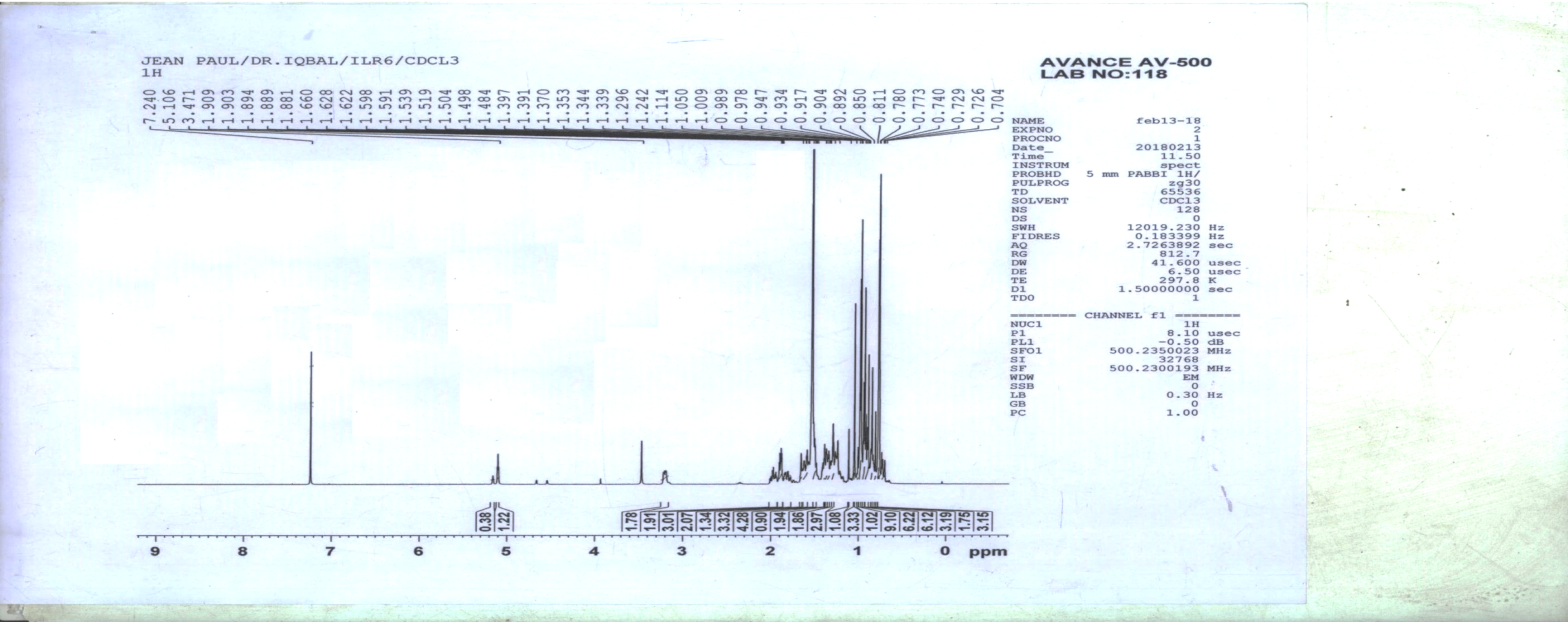


**Figure S10:** ^1^H NMR spectrum (CDCl_3_, 400 MHz) of *α*-amyrin (3β-hydroxy-urs-12-en-3-ol) (**5**)


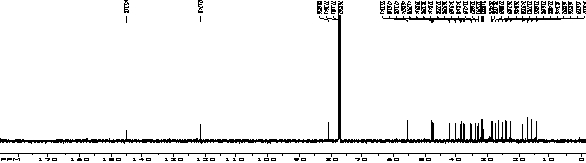


**Figure S11:** ^13^C NMR spectrum (CDCl_3_, 100 MHz) of *α*-amyrin (3β-hydroxy-urs-12-en-3-ol) (**5**)


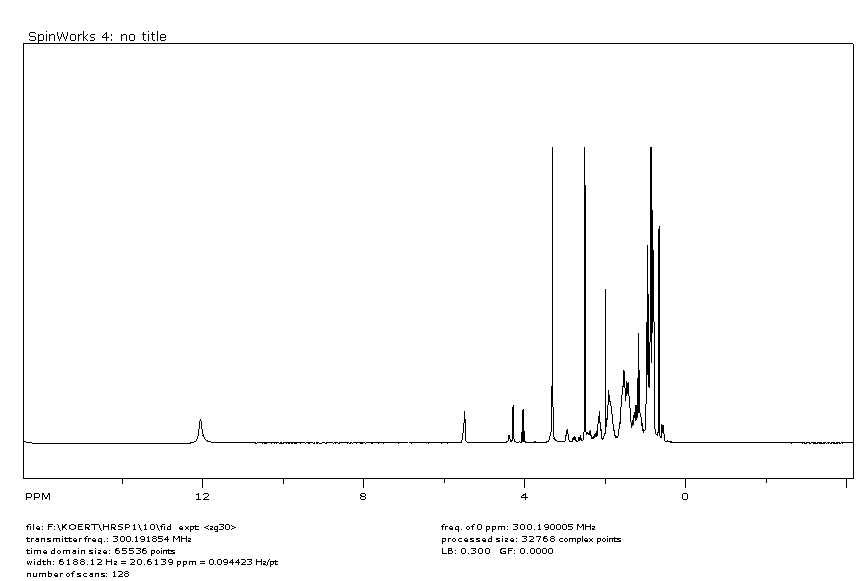
 **Figure S12-1:** ^1^H NMR spectrum (DMSO-*d_6_*, 400 MHz) of quinovic acid 3-O-[ *α*-D-quinovopyranoside] (**6**).


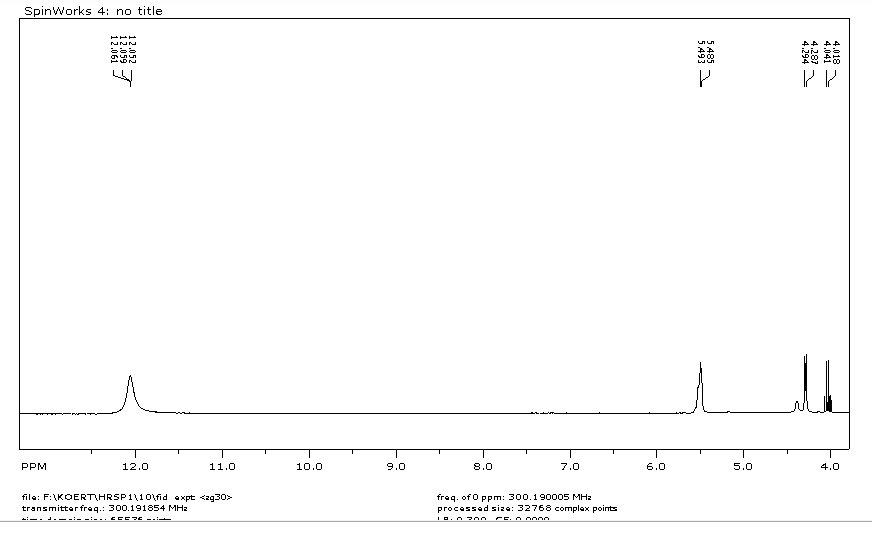


**Figure S12-2:** ^1^H NMR spectrum (DMSO-*d_6_*, 400 MHz) of quinovic acid 3-O-[ *α*-D-quinovopyranoside] (**6**).


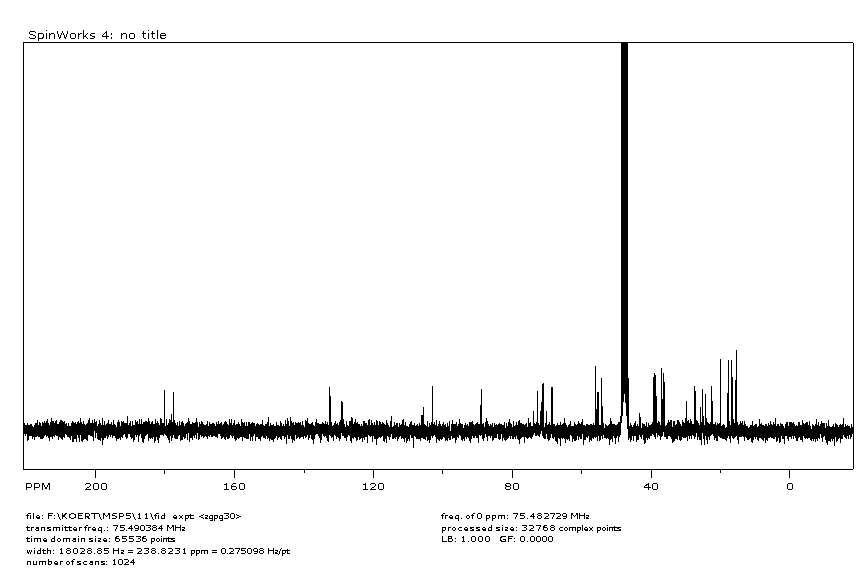


**Figure S13:** ^13^C NMR spectrum (DMSO-*d_6_* , 100 MHz) of quinovic acid 3-O-[ *α*-D-quinovopyranoside] (**6**).


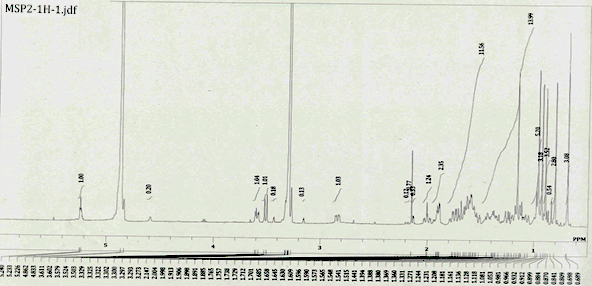


**Figure S14:** ^1^H NMR spectrum (CDCl_3_, 400 MHz) of erythrodiol (**7**).


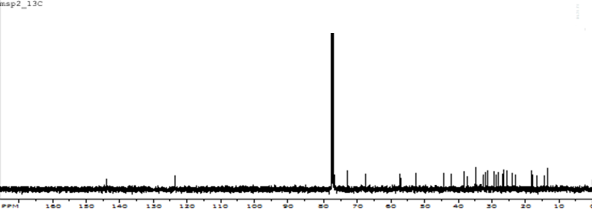


**Figure S15:** ^13^C NMR spectrum (CDCl_3_, 100 MHz) of erythrodiol (**7**).

**
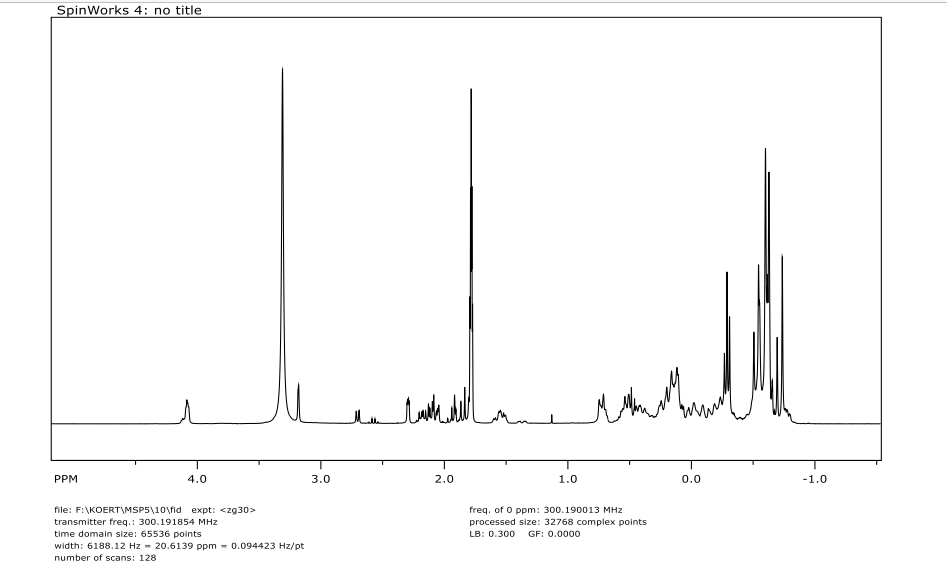
**

**Figure S16:** ^1^H NMR spectrum (DMSO-*d_6_*, 400 MHz) of quinovic acid (**8**).


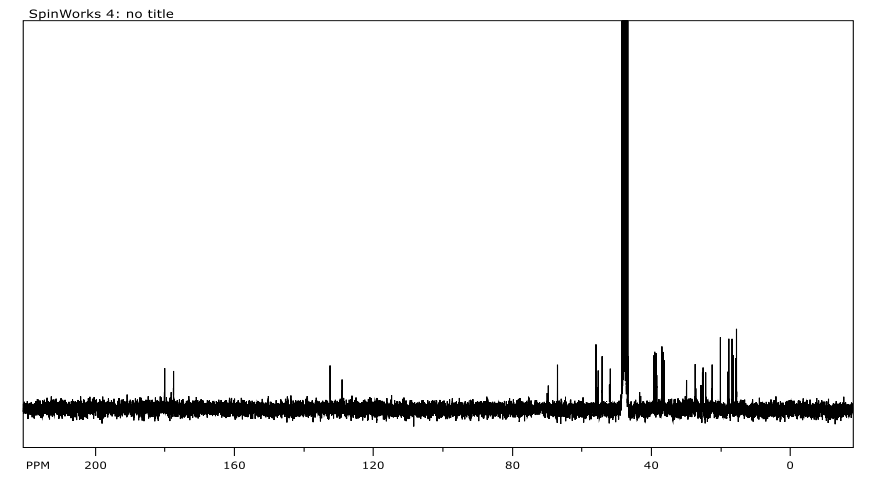


**Figure S17:** ^13^C NMR spectrum (DMSO-*d_6_*, 100 MHz) of quinovic acid (**8**).


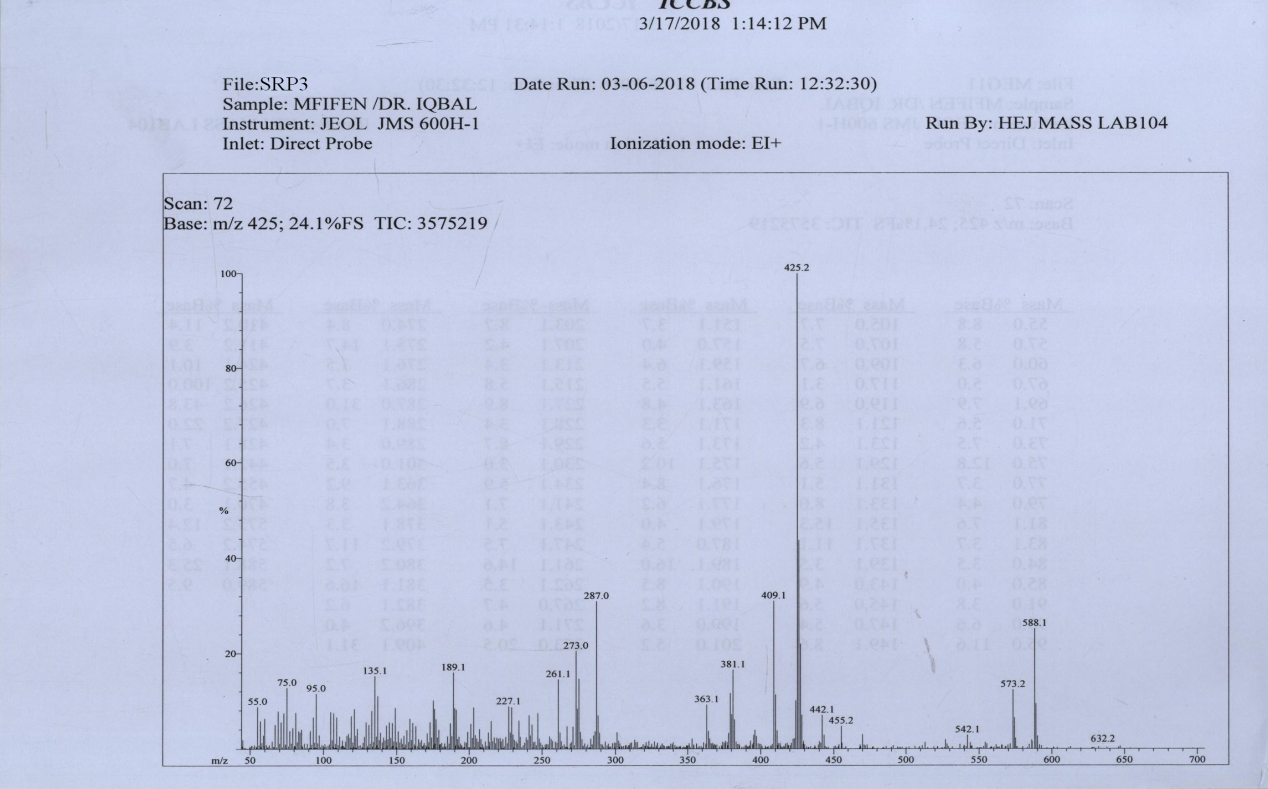


**Figure S18:** Mass spectrum of quinovic acid 3-O-[β-D-quinovopyranoside] (**9**).


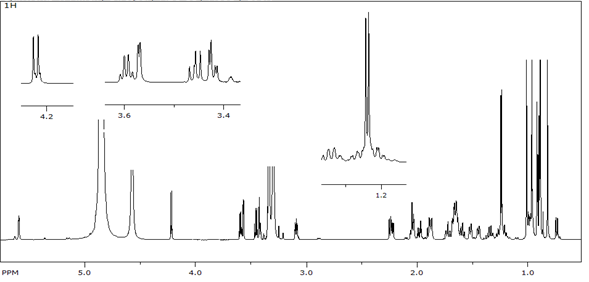


**Figure S19:** ^1^H NMR spectrum (MeOH-*d_4_*, 500 MHz) of quinovic acid 3-O-[β-D-quinovopyranoside] (**9**).


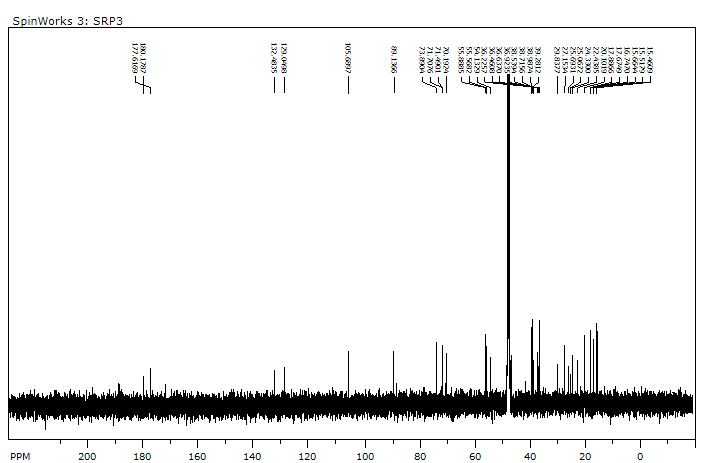


**Figure S20:** ^13^C NMR spectrum (MeOH-*d_4,_* 125MHz) of quinovic acid 3-O-[β-D-quinovopyranoside] (**9**).

**
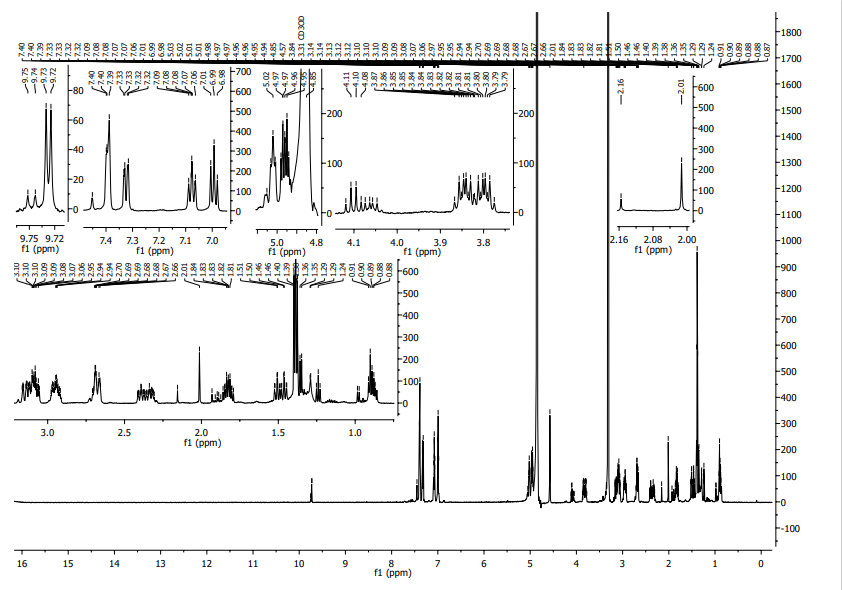
**

**Figure S21:** ^1^H NMR spectrum (CD3OD, 400 MHz) of latifoliamide C (**10**).

**
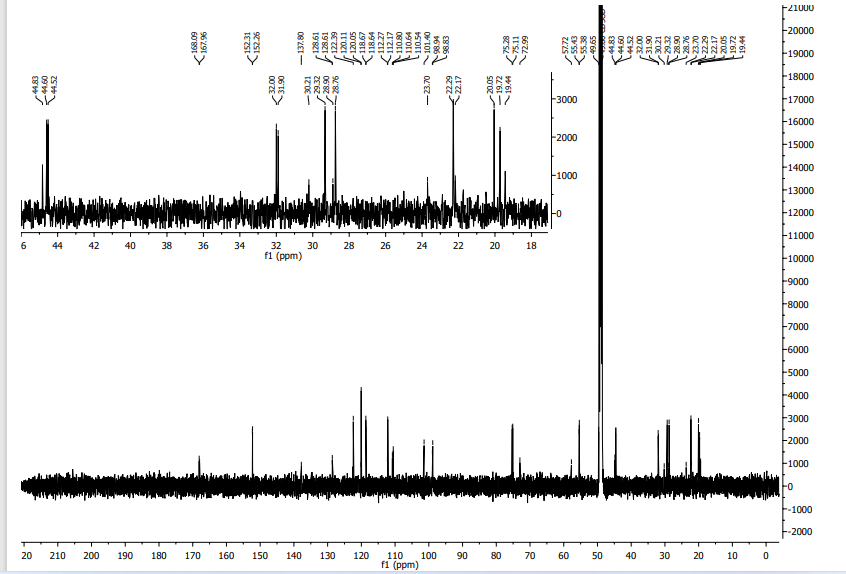
**

**Figure S22:** ^13^C NMR spectrum (CD3OD, 100 MHz) of latifoliamide C (**10**).
